# Supplementary material for: Fluctuations in AKT and PTEN Activity Are Linked by the E3 Ubiquitin Ligase cCBL
Source: Cells. 2021 Oct 20;10(11):2803. doi: 10.3390/cells10112803 (PMC8616390; doi:10.3390/cells10112803)
Supplement: Supplementary file 1 [file cells-10-02803-s001.zip › supplemental information and videos/3 REB Olazabal Supplemental informationv2.pdf]

Oscillation of AKT and PTEN activities are linked by the E3 ubiquitin ligase cCBL

Manuel Olazábal-Morán[1], Miriam Sánchez-Ortega[1], Laura Martínez-Muñoz[2,3], Carmen Hernández[1], Manuel S. Rodríguez[4], Mario Mellado[1] and Ana C. Carrera[1*]

Supplementary INFORMATION including:

Legends to Supplementary videos 1 to 4.

Supplementary Materials and Methods.

Supplementary Figure 1 to 8.

Legends to Supplementary videos 1 to 4 (videos submitted independently).

Supplementary video 1: GFP control signal in FCS-activated NIH3T3 cells. NIH3T3 cells expressing CONTROL GFP vector were incubated in serum-free media (2h) and then activated with PDGF (50ng/ml). The movie shows the image frames collected every 2 minutes (for 90 minutes).

Supplementary video 2: GFP control signal in PDGF-activated NIH3T3 cells. NIH3T3 cells expressing CONTROL GFP were serum-starved (2h) and activated with FCS (15%). Image frames were collected every 2 minutes (for 90 minutes).

Supplementary video 3: PIP3 membrane signal in FCS-activated NIH3T3 cells. NIH3T3 cells expressing pEGFP-Btk-PH were incubated in serum-free media (2h) and then activated with PDGF (50ng/ml). The movie shows the image frames collected every 2 minutes (for 90 minutes).

Supplementary video 4: PIP3 membrane signal in PDGF-activated NIH3T3 cells. NIH3T3 cells expressing pEGFP-Btk-PH were serum-starved (2 h) and activated with FCS (15%). Image frames were collected every 2 minutes (for 90 minutes).

Supplementary Materials and Methods:

Fluorescence resonance energy transfer experiments. HEK-293T (3 x 10^5 cells/well) were transfected with cDNA as above (48h). For the p85α/p110α curves, CFP-p85α (0.4 μg) and increasing amounts of YFP-p110α (0.2-3 μg) were used; as control, a constant amount of CFP-SH3α (0.2 μg) and increasing amounts of YFP-p110α (0.2-3 μg) were used. p85β/p110β heterodimers were examined similarly but using YFP-p110β and CFP-SH3β (0.2 μg) as controls. For p110α/p110β complexes, a constant amount (0.5 μg) of a mixture of CFP-p110α and pSG5-HAp85α (1:0.2) and increasing amounts of the mix YFP-p110β plus pSG5-HAp85β; 1:0.4) were used. Alternatively, a constant amount of CFP-p110β:pSG5-HAp85β (0.5 μg; 1:0.4) and increasing quantities (0.2-to-2.5 μg) of YFP-p110α and pSG5-HAp85α (1:0.2) were used. For control, a constant amount (0.5 μg) of CFP-p110α andpSG5-p50α (1:0.2) and increasing amounts (0.2-2.5 μg) of YFP-p110β:pSG5-p50α (1:0.4) were used.

At harvest, cells were suspended in HBSS plus 0.1% glucose. A cell suspension equivalent to 20 μg protein (in 100 μl/well) was placed in black 96-well plates and light emission was quantified using a Wallac Envision 2104 Multilabel Reader (PerkinElmer) equipped with a high-energy xenon flash lamp (for CFP 8 nm bandwidth excitation filter 405 nm, for YFP 10 nm bandwidth excitation filter 510 nm). Gain settings were identical in all experiments. To determine the spectral signature, cells were transiently transfected with the proteins fused to CFP or YFP. The contribution of CFP or YFP alone was measured in each detection channel, and normalized to the sum of both [37]. The spectral signatures of the CFP- or YFP-proteins did not vary significantly (p>0.05) from the signatures determined for each fluorescent protein. For the quantitation of FRET in saturation curves and YFP-protein expression, the spectral signature was used in linear unmixing to separate the two emission spectra. The fluorescence emitted by each fluorophore in FRET was determined using the formulae: CFP=S/(1+1/R) and YFP=S/1+R, where S=ChCFP + ChYFP, R= $(YFP_{530}Q - YFP_{510})/(CFP_{510} - CFP_{530}Q)$ and Q=ChCFP/ChYFP. ChCFP and ChYFP represent the signal for the 510 nm and 530 nm

detection channels (Ch); CFP_{510}, CFP_{530}, YFP_{530} and YFP_{510} represent the normalized contributions of CFP and YFP to channels 510-530, as determined from the spectral signatures of the fluorescent proteins. The $FRET_{50}$ and $FRET_{max}$ values were obtained by extrapolating data using a non-linear regression equation in a single binding site model (95% confidence interval) (GraphPad PRISM). Specific software was generated in MatLab (The MathWorks Inc) to determine whether the FRET saturation curves ($n \geq 3$) were similar (accept H_0) or significantly different (reject H_0); this was done using the F test, Bootstrap and AICc tests [38].

Bioluminescence resonance energy transfer. To prepare BRET titration curves, HEK-293T (6 x 10^5) cells were transiently cotransfected with a constant amount of cDNA encoding PTEN-Rluc (0.3 μg), plus increasing amounts of YFP-p85α (0.8 -0.1 μg). Fluorescent proteins were quantified using coelenterazine H (5 μM, 1 min, RT) in a Wallac Envision 2104 Multilabel Reader equipped with a high-energy xenon flash lamp (10 nm bandwidth excitation filter at 510 nm) as described [39]. PTEN-Rluc luminescence signals were acquired 10 min after the addition of coelenterazine H. BRET efficiency ($BRET_{eff}$) is defined as [(long wavelength emission)/(short wavelength emission)]-Cf, where Cf is [(long wavelength emission)/(short wavelength emission)] for the Rluc construct expressed alone. For each BRET titration curve, the relative amount of acceptor is given by the ratio between acceptor fluorescence (YFP) and donor luciferase activity (Rluc). The $BRET_{50}$ and $BRET_{max}$ values were obtained by extrapolating data in a non-linear regression equation (95% confidence interval) in a single binding site model. The $BRET_{50}$ and $BRET_{max}$ values in these BRET saturation curves (n = 3) were then analyzed. For fixed ratio BRET, HEK-293T cells (9 x 10^6) were co-transfected with PTEN-Rluc (4.0 μg) and YFP-p85α (6.4 μg) (48h). Cell were incubated in serum free medium (2 h), detached with HBSS + 0.1% glucose (37ºC), and placed in 96-well microplates (0.3 μg/μl, 90 μl). Coelenterazine H was then added (5 μM, 100 μl/well) and readings taken (as above) after EGF (100 ng/ml) addition (determinations were obtained at 0.05 s intervals). BRET signal expressed in BRET units (BU).

Primers List. For pRK5-PTEN-GST construction, primers for the PTEN stop codon were: 5′GCATACACAAATTACAAAAGTCGGATAAGTCGACCTCGAGG3′,5′CGTCGAGGTCGACTTATCCGAC TTTTGTAAATTTGTGTATGC 3′. The primers for GST-PTEN PCR were 5′GACTGTCGACAT GTCCCCTATACTAGGTTATTGGAAAATTAAGGGCC 3′& 5′GATCGGGCCCCGTCAGTCAGTCACGA TGAATTCCCGG 3′; for pLuc-PTEN they were 5′TAGAATTCATGACAGCCATCATCAAAGAG3′ & 5′GGGATCCACTTTTGTAATTTGTGTATGC3′; for UBC12 5′AGTTTTAAGGTGGGCCAGG 3′ & 5′TCGAGGTCAATGTTGGGGTG3'; and for GAPDH 5′GAAGGTGAAGGTCGGAGTC3′ & 5′GAAGATGGTGATGGGATTTC3'. For CFP/YFP-p110α 5′TCTCGAGCCATGGAACAGAAAC TCATATCG3′,5′ & GGATCCTCAGTTCAAAGCATGCTGCTTAATTG 3′, for CFP/YFP-p110β 5′ TTCTCGAGCCATGGAGCAGAAGCTGATTTCC 3′ & 5′GAATTCTTAAGATCTGTAGTCTTTCCGA AC3′, for CFP/YFP-p85α 5′ TTTGAATTCTACCATGTACCCGTACGATGTC 3′ & 5′ GGATCCTCA AGTCTCAGGCTC 3′, -p85β 5′ TTTGAATTCTATGGCGGGCCCTGAGGGC 3′ & 5′ GGTACCTCA GCGGGCGGCAGGCGG3′, -SH3 (p85α) 5′TTTGAATTCTACCAT GTACCCGTACGATGTC 3′ & 5′ GGTACCTCAGTTGTTGGCTACAGTAGTGGG 3′, and -SH3(p85β) 5′ TTTGAATTCTATGGCGGGC CCTGAGGGC 3′ & 5′ GGTACCTCACCCTCCATTGGCCAGGACTG 3′.

Antibody List. Ab for WB were: CT-PTEN (138G6, 9559), pPTEN (44A7, 9549), Thr^{308}pAKT (C31E5E, 2965), Ser^{473}pAKT (9271), AKT (9272), p110β (C33D4, 3011), Ser^{241}pPDK1 (3061), PDK1 (D37A7, 5662), and EGFR (D38B1, 4267) from Cell signaling technology. Ab for pan-p85 (06-195), p85-N-SH3 (AB6, 05-212), core-histones (13-107) and NEDD4 (2740) came from Millipore. Ab for p85β (1C8) (18), β-actin (A5441) from Sigma, α-tubulin (CP06) from Calbiochem, SUMO-1 (Y299, ab32058) and SUMO-2/3 (b3742) from Abcam, and for ubiquitin (P4D1, sc-8017) and NT-PTEN (sc-6818) from Santa Cruz Biotech. Ab to CBL-b (NBP1-44920) were from Novus, and for cCBL (610442) from BD Biosciences. For IP, anti-CT-PTEN Ab (138G6, 9559), PTEN XP bead conjugate (D4.3, 4326), rabbit mAb XP Isotype Control (DA1E, 3423) and EGFR (D38B1, 4267) were from CTS; for p85α (05-212), pan-p85-N-SH3 (05-212) from Millipore, and for cCBL (17/cCBL, 610442) from BD Biosciences.

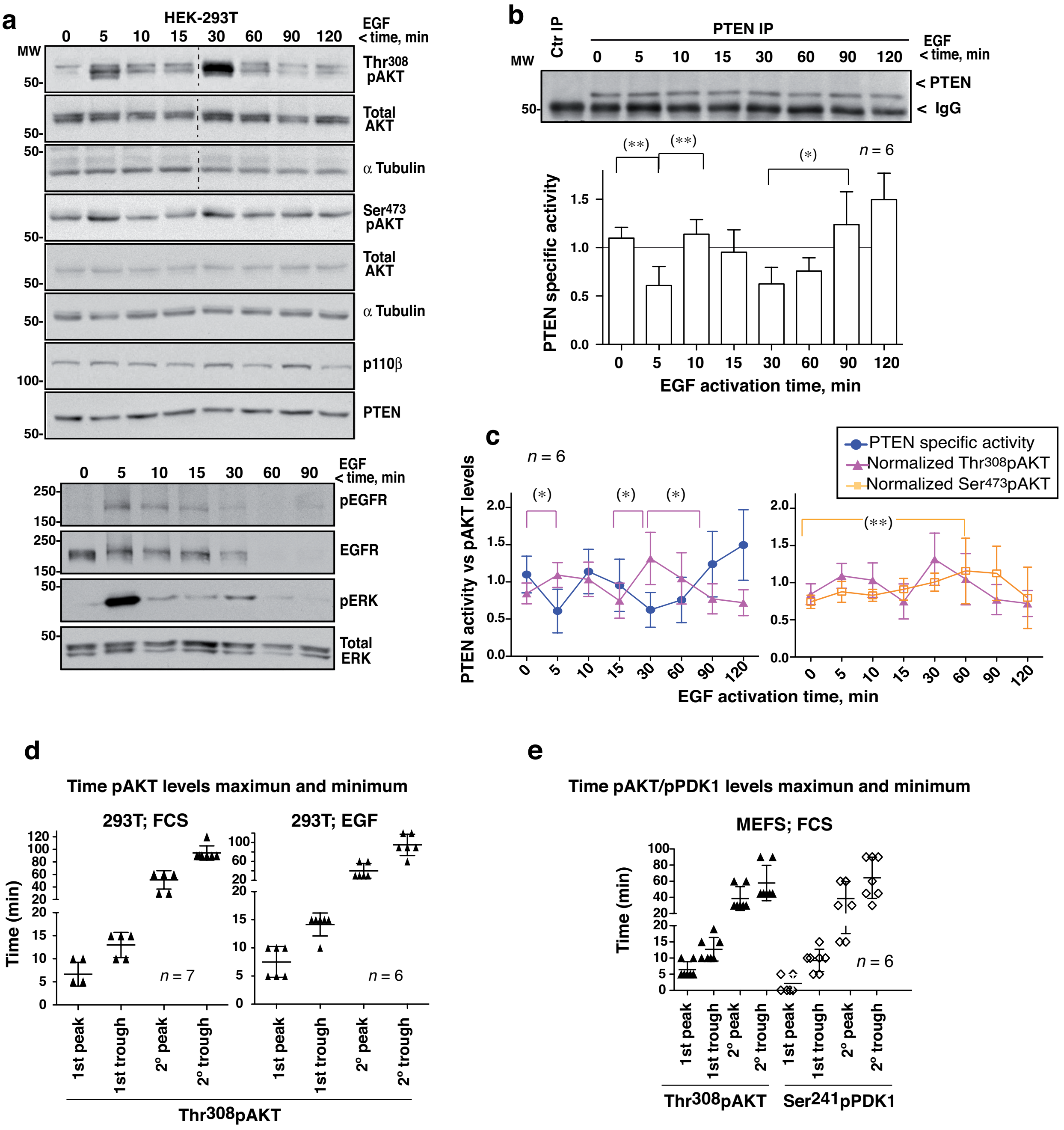

Supplementary Figure S1: EGF addition leads to complementary changes in AKT and PTEN activity. (**a-d**) HEK-293T cells were incubated in serum-free media (2 h) then activated with serum 15% (a) or EGF (100 ng/ml) (b-d) for different times (indicated). Cells were lysed in RIPA and proteins analyzed by WB (indicated) (a). (**b**) PTEN was immunopurified from cell extracts (1 mg). The immunoprecipitated samples were then incubated in phosphatase buffer containing PIP_3. The PIP_2 product was quantified as in main figure 1. (**c**) pAKT signals (from assays as in b) were measured and corrected to AKT and β-Actin levels. Corrected pAKT signals were normalized to the pAKT signal average (considered 1) and represented with PTEN activity levels (mean ± SD, n = 6). (**d**,**e**) The graphs represent the mean ± SD times at which the first and second pAKT (or p-PDK1) peaks and valleys were detected in HEK-293T (d) or MEF (e), GF are indicated. Dashed lines indicate spliced time points (see originals in supplementary information). *P<0.05, **P<0.01 Student paired t test.

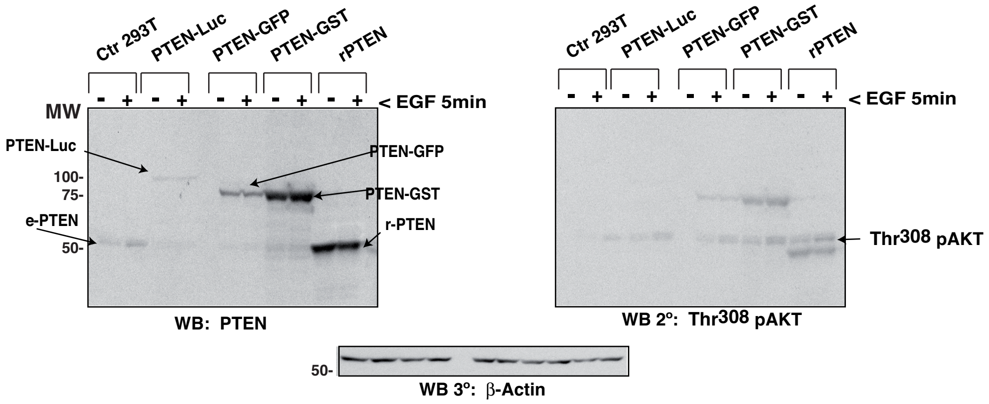

Supplementary Figure S2: PTEN expression levels in HEK-293T cells. HEK-293T cells were transfected with different cDNAs (48h) (indicated), incubated without serum (2 h) and then treated with EGF (100 ng/ml) for 5 min. PTEN expression and pAKT levels (indicated) were examined in WB.

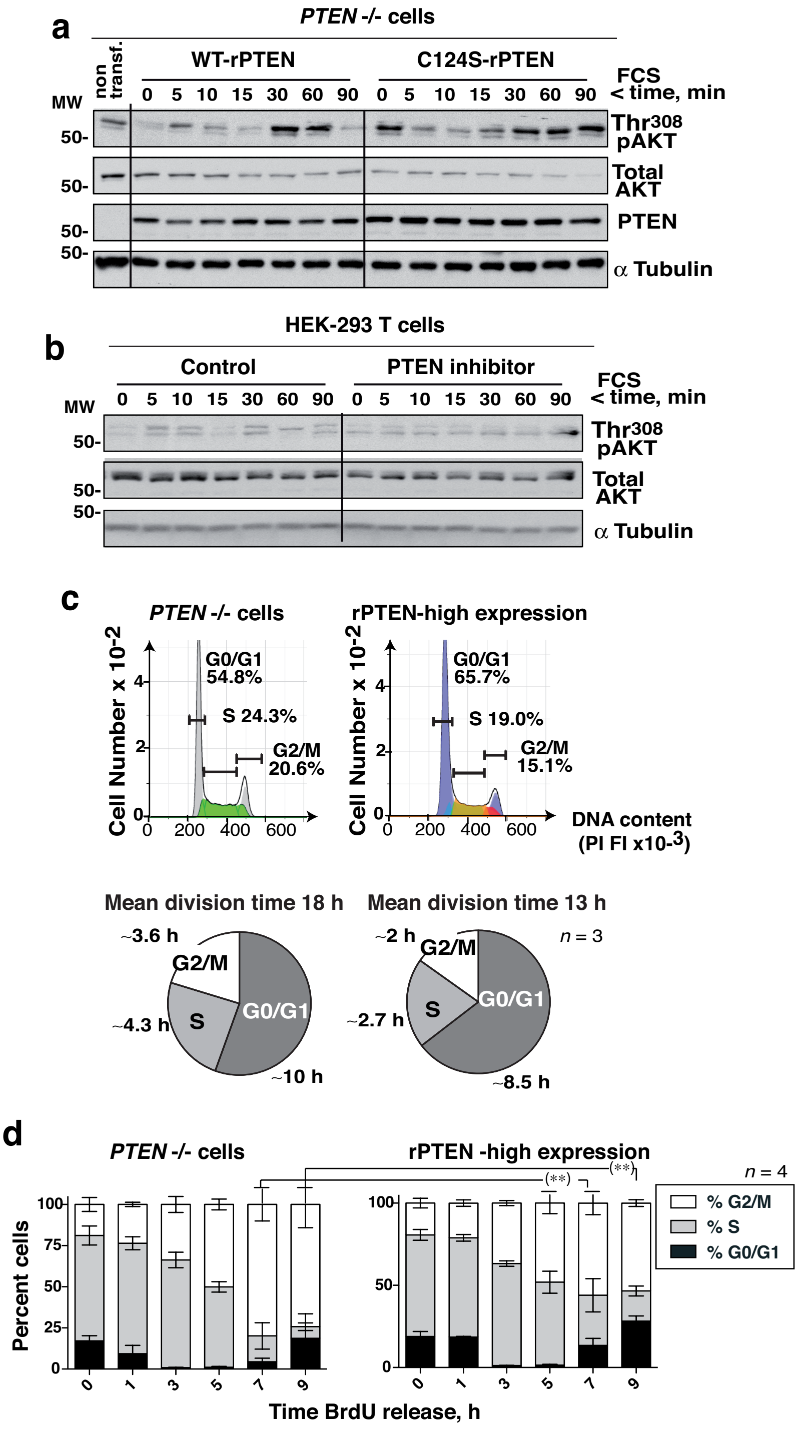

Supplementary Figure S3: PTEN reconstitution in *PTEN* null cells accelerates cell division. (**a**) PC3 cells were transfected with control, pRK5-WT-PTEN or pRK5-C124S-PTEN (48 h). Cells were then incubated in medium without FCS (2 h), and activated with FCS (15%) for different times. Blots show Thr308pAKT and PTEN levels in the different conditions (indicated). (**b**) DNA content of exponentially growing control or pRK5-PTEN-reconstituted PC3 cells. Mean division time (*n* = 3) was calculated by seeding a fixed number of cells and counting them every 12 h. Circular graphs represent the time spent in each cell cycle phase. (**c**) PC3 cells transfected with control vector or pRK5-PTEN cDNA (48 h) were labeled with BrdU for 1 h, then BrdU deprived and incubated in normal media for different times. Distribution of cells in the different cell cycle phases, mean ± SD, *n* = 4. **P<0.01 Chi Squared test.

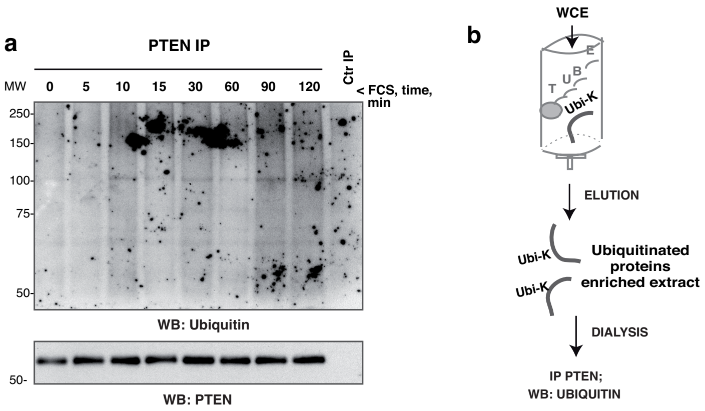

Supplementary Figure S4: Cell activation with FCS triggers intermittent PTEN ubiquitination. (**a**) PTEN was immunoprecipitated from extracts of HEK-293T cells activated with FCS for different times and examined by WB. Secondary blots confirmed IP efficiency. (**b**) The diagram shows the experimental protocol for concentration of ubiquitinated proteins in TUBES-columns. Upon elution, ubiquitin-enriched extracts were dialyzed and used for PTEN IP; the analysis of PTEN ubiquitination was by WB.

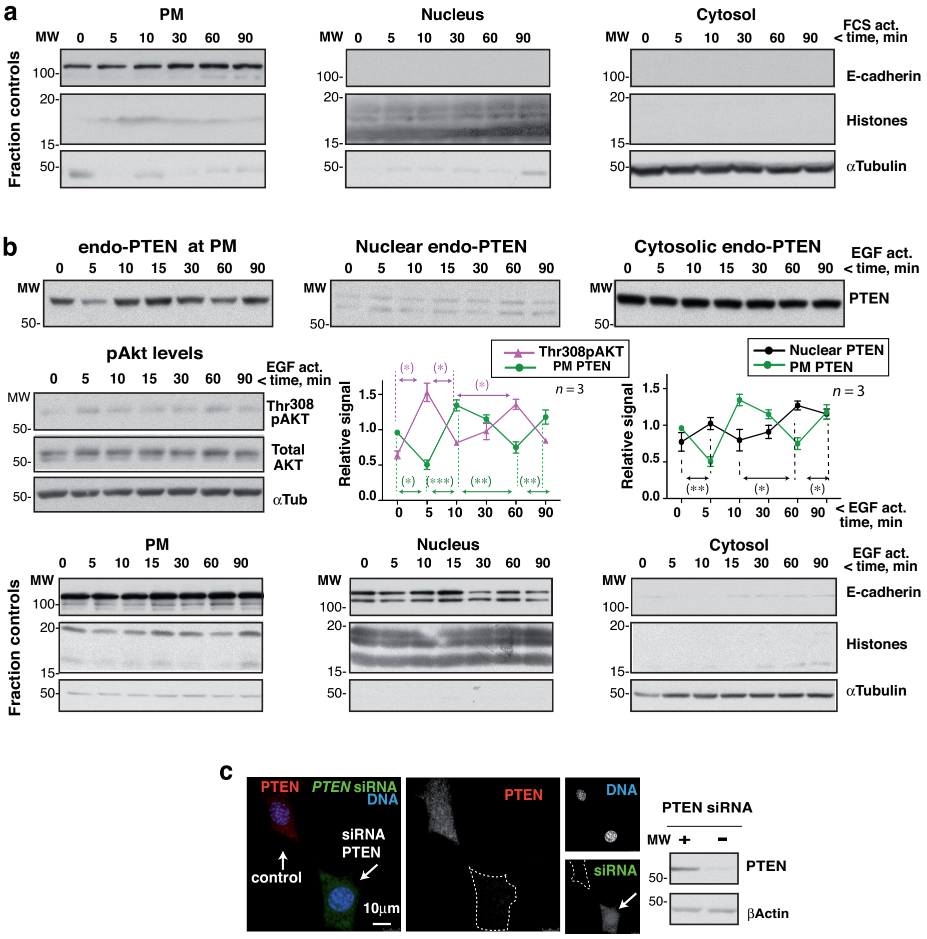

Supplementary Figure S5: pAKT oscillation co-occurs with changes in PTEN localization. (**a**) Cell fractionation controls for main figure 5a. (**b**) HEK-293T cells were incubated in serum-free media (2 h), stimulated with EGF (100 ng/ml) and examined as in main Figure 5. The graphs were as in main Figure 5 (mean ± SD, n = 3). *P<0.05, **P<0.01, ***P<0.001 paired Student t test. (**c**) NIH3T3 cells were co-transfected with GFP and Pten-specific siRNA (72 h), then examined by IF using anti-PTEN Ab (red), Hoechst 33258 (DNA, blue) and GFP signal (green). GFP marks the cells transfected with siRNA; PTEN depletion was confirmed in WB.

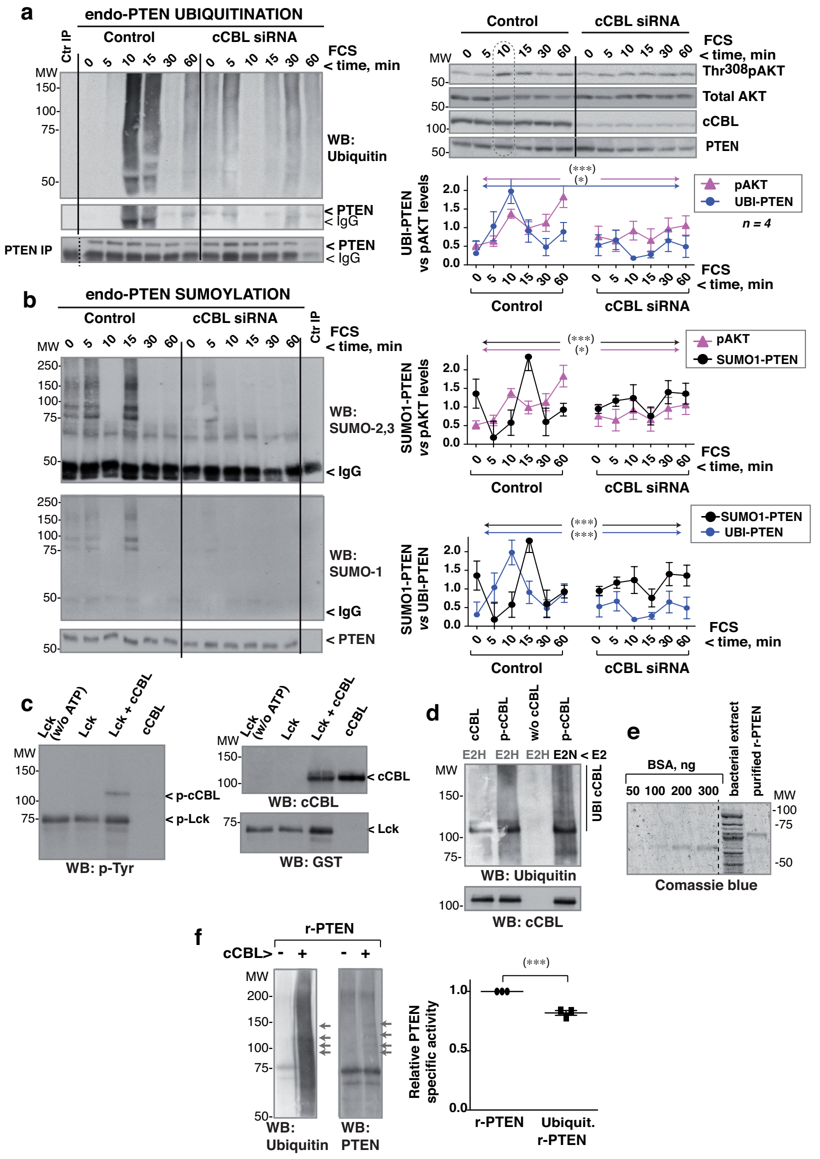

Figure S6: *In vitro* ubiquitination of PTEN by cCBL. (**a**) HEK-293T cells were transfected with control or cCBL siRNA (72 h). Cells were processed and graphs constructed as in main figure 6a; graphs show the mean ± SD (of *n*=4 with first pAKT peak at 10 min). For normalization, a fraction of the cells were lysed in RIPA and extracts used for PTEN IP and immunoblot (indicated). (**b**) RIPA extracts of cells as in (a) (1 mg) were incubated with anti-PTEN Ab beads and examined in WB using anti- SUMO2/3 or SUMO1 Ab. Secondary blots confirmed PTEN IP efficiency. For controls (Ctr IP), extracts were incubated with control beads. SUMO1-PTEN signal (70-100 KDa) was corrected for PTEN cellular levels, and referred to the SUMO1-PTEN signal average in controls (considered 1). The graphs show sumoylated-PTEN signals compared to normalized pAKT levels (top) or ubiquitinated-PTEN levels (bottom). (a, b) *P<0.05; ***P<0.001 Chi Squared test. (**c**) HEK-293T NP40 cell extracts (1 mg) were used for cCBL IP and these subjected to phosphorylation using Y505F-Lck. The reaction was examined in WB. Purified Y505F-Lck is auto-phosphorylated in bacteria even prior to the kinase assay as reported [18], but cCBL only upon the kinase reaction. (**d**) cCBL and p-cCBL (as in c) were incubated with ubiquitin, E1 (200ng) and E2 (250 ng) ligases (indicated) and the reactions examined by WB. (**e**) Coomassie blue staining of bacterially purified GST-PTEN (r-PTEN); the first lanes include BSA. (**f**) Phosphatase activity of r-PTEN prior to and upon cCBL-mediated ubiquitination, as tested in WB. Graphs show the PTEN phosphatase activity from three assays (mean ± SD, n = 3); in each assay, normalized PIP_2 levels were referred to those of control PTEN (not ubiquitinated), considered 1. ***P<0.001 Student unpaired *t* test.

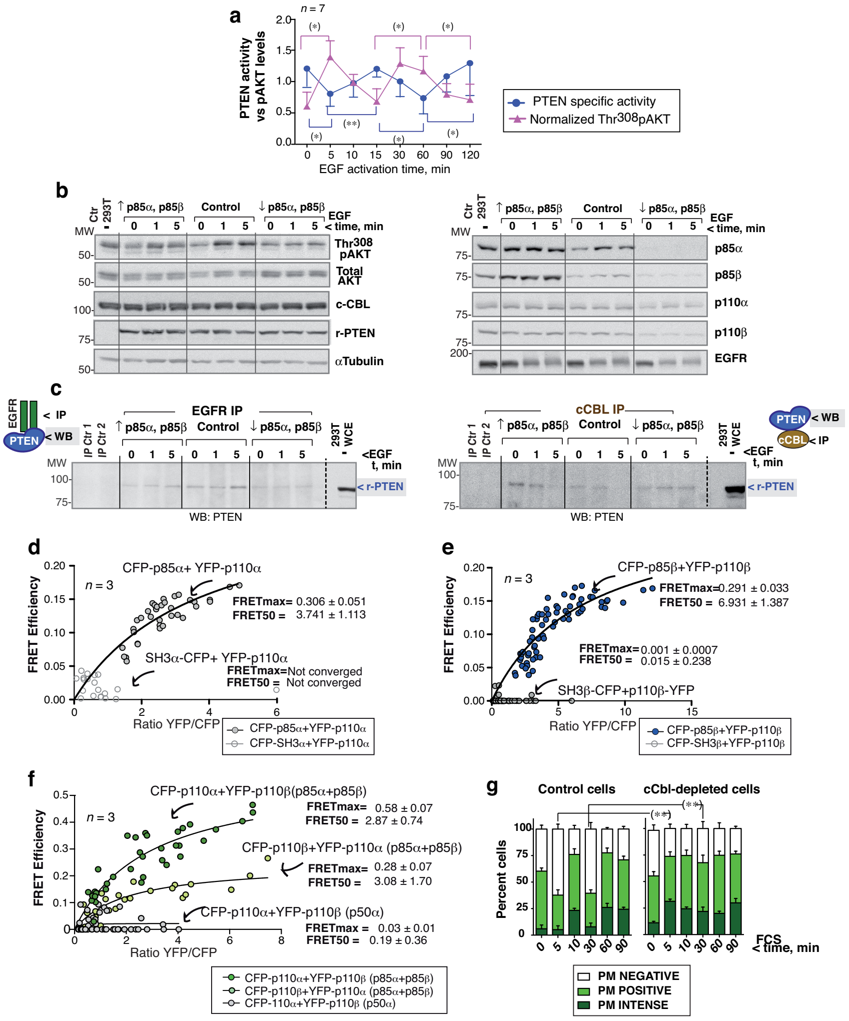

Figure S7. Optimization of resonance energy transfer assays. (**a**) HEK-293T cells transfected with PTEN-Luc (48 h) were activated by the addition of EGF (100 ng/ml) for different times. pAKT levels in extracts were examined in WB. pAKT signals were quantitated and represented as in main figure 1. Extracts (1 mg) were also used for PTEN IP and phosphatase activity examined and represented as in main figure 1. The graphs represent the relative PTEN activity and the Thr308pAKT levels at each time point (mean ± SD, n = 7). *P<0.05, **P<0.01, Student paired t test. (**b**) HEK-293T cells were transfected with PTEN-Luc (control) or with PTEN-Luc, HA-p85α and HA-p85β (48h) (indicated as ↑p85α, p85β) or with *PIK3R1* plus *PIK3R2* siRNA (72 h), and then PTEN-Luc (last 48h) (↓p85α, p85β); extracts were examined in WB. (**c**) HEK-293T cells were transfected, collected and processed with the indicated Ab as in main figure 7b. cCBL in complex with EGFR and PTEN in complex with cCBL were tested by WB. Blots to control IP efficiency appear in main Figure 7. (**d-f**) FRET saturation curves were generated using HEK-293T cells transiently transfected with a constant amount of CFP-p85α or -p85β, and increasing amounts of YFP-p110α or -p110β (indicated). For controls, cells were co-transfected with a constant amount of CFP-SH3α or -SH3β and increasing amounts of YFP-p110α (d) or -p110β (e). In (**f**), FRET saturation curves were generated using HEK-293T cells transfected with a constant amount of CFP-p110α:(HAp85α + HAp85β) and increasing amounts of YFP-p110β or, alternatively, with a fixed amount of CFP-p110 β :(HAp85α + HAp85β) and increasing amounts of YFP-p110α. A constant amount of CFP-p110α+p50α and increasing amounts of YFP-p110 β was used as control. (d-f) Plots indicate FRETmax/BRETmax (mean ± SD) as calculated using a non-linear regression equation for a single binding-site model (95% confidence interval; n = 3). (**g**) NIH3T3 cells were transfected with mouse-specific cCbl siRNA mix, or with control siRNA. After 72h, cells were transferred to glass coverslips and incubated for an additional 24 h. Cells were then incubated in serum free media (2 h) and incubated with FCS for different times. The graphs show the percentage of cells with a PTEN PM intense, positive, or negative signal at different times after FCS addition (mean ± SD, n >100). **P<0.01 Chi Squared test.
