# Supplementary material for: Fluctuations in AKT and PTEN Activity Are Linked by the E3 Ubiquitin Ligase cCBL
Source: Cells. 2021 Oct 20;10(11):2803. doi: 10.3390/cells10112803 (PMC8616390; doi:10.3390/cells10112803)
Supplement: Supplementary file 1 [file cells-10-02803-s001.zip › supplemental information and videos/original_images.v2.pdf]

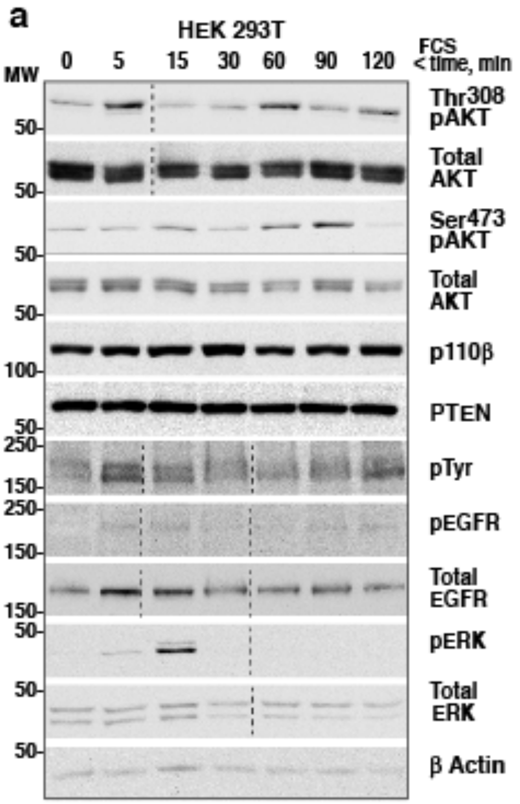

Fig1a:

1ºT308 pAKT

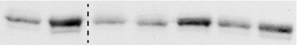

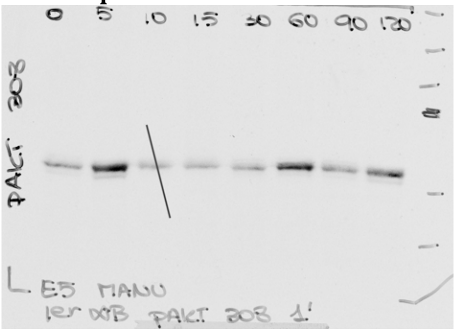

2ºTOTAL AKT

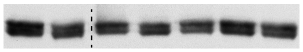

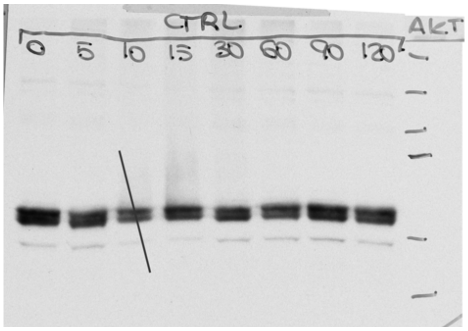

3ºSer 473 pAKT 473pAKT was examined in a different gel; 10 min was not examined **4ºTOTAL AKT**

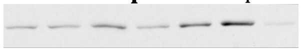

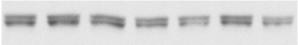

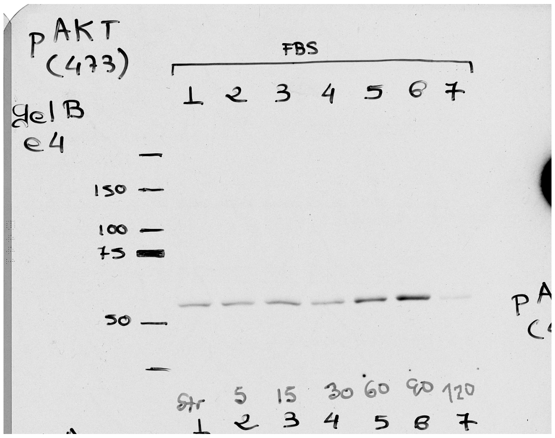

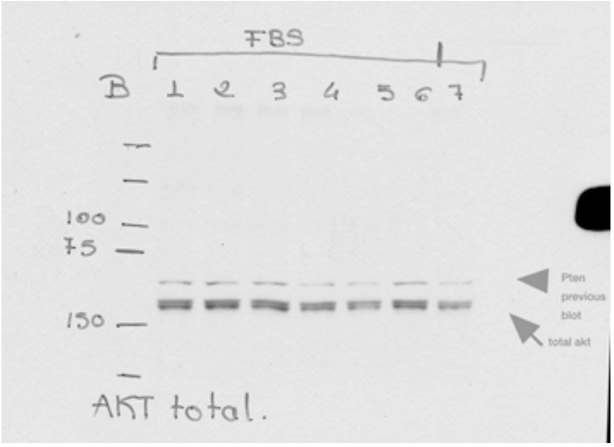

5ºp110β

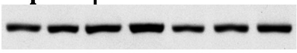

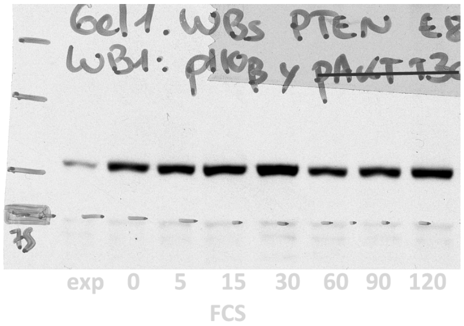

6ºPTEN

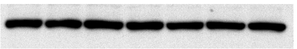

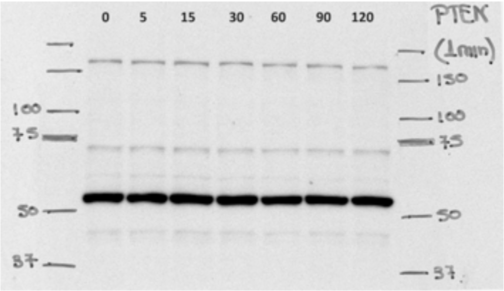

Supplemental FigS1a

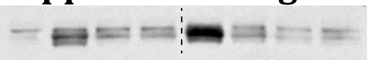

1ºT308 pAKT

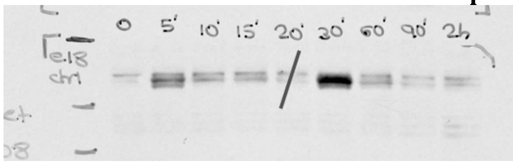

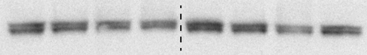

2ºTOTAL AKT

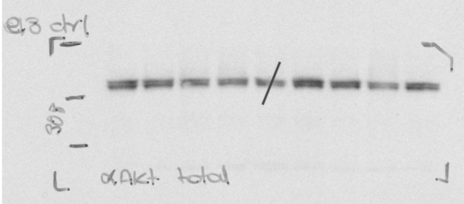

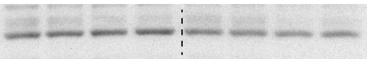

3ºαTubulin

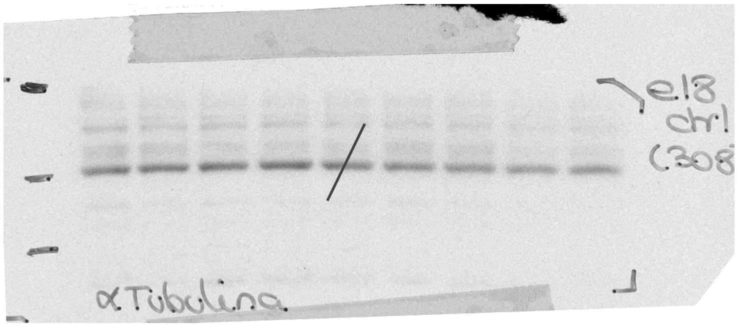

4ºSer 473 pAKT

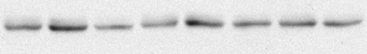

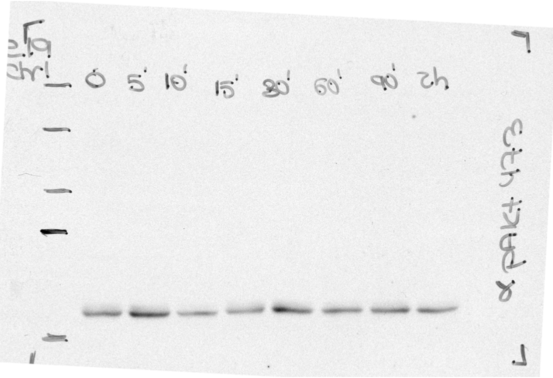

5ºTOTAL Ser 473 pAKT

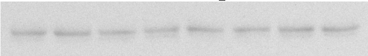

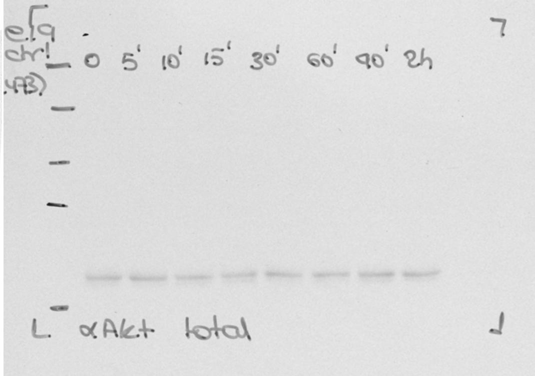

6ºαTubulin

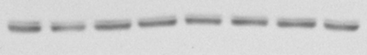

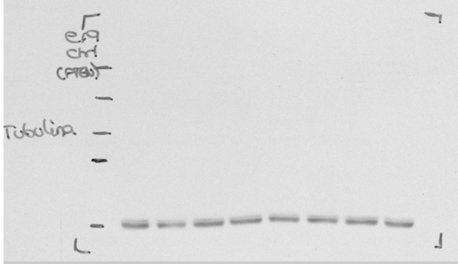

7ºp110β p110beta was resolved in a different gel

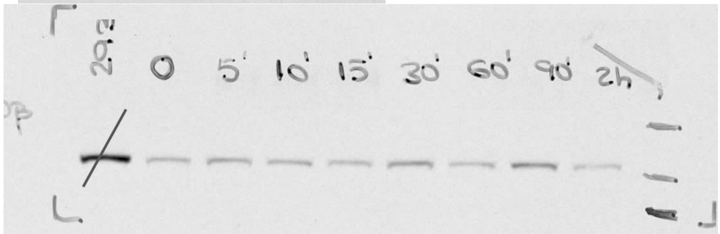

8ºPTEN

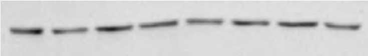

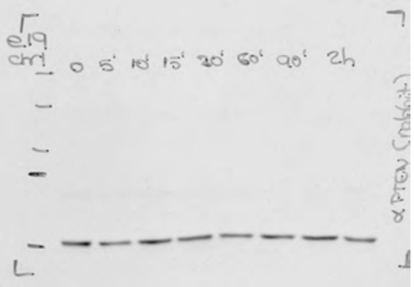

FigS1a bottom

1ºp-EGFR

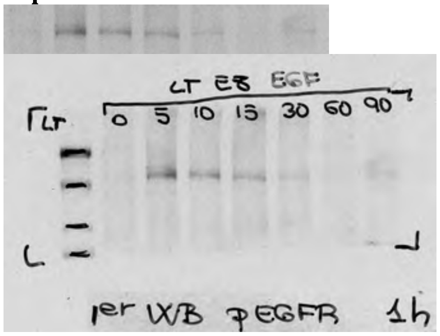

2º EGFR

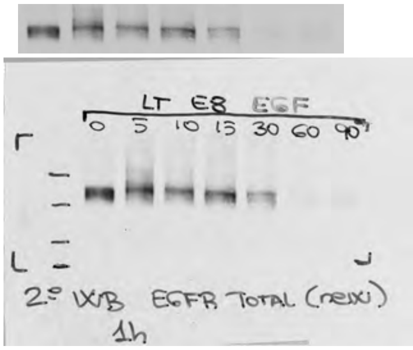

3ºp-ERK

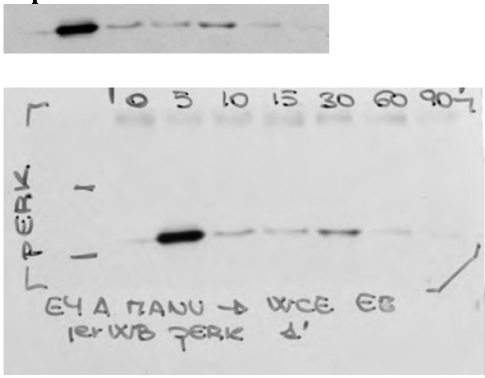

4º Total ERK

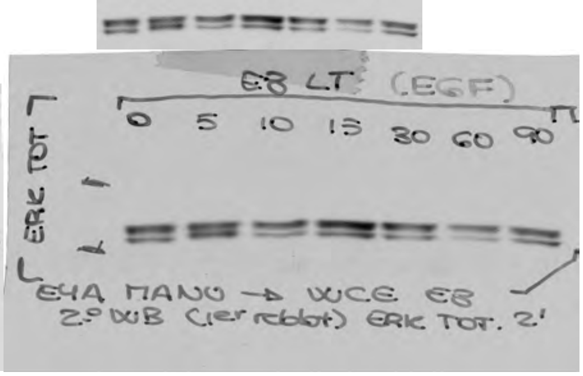

FigS1b e1b kinetics EGF

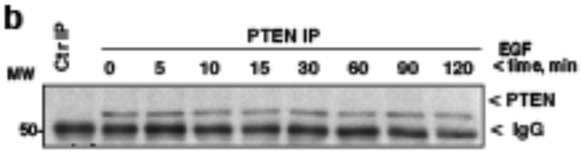

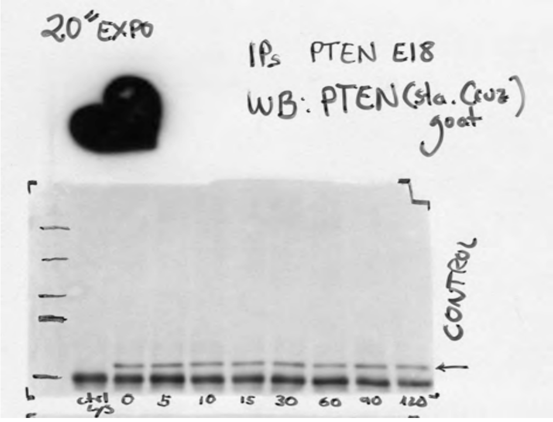

Supplemental FigS2

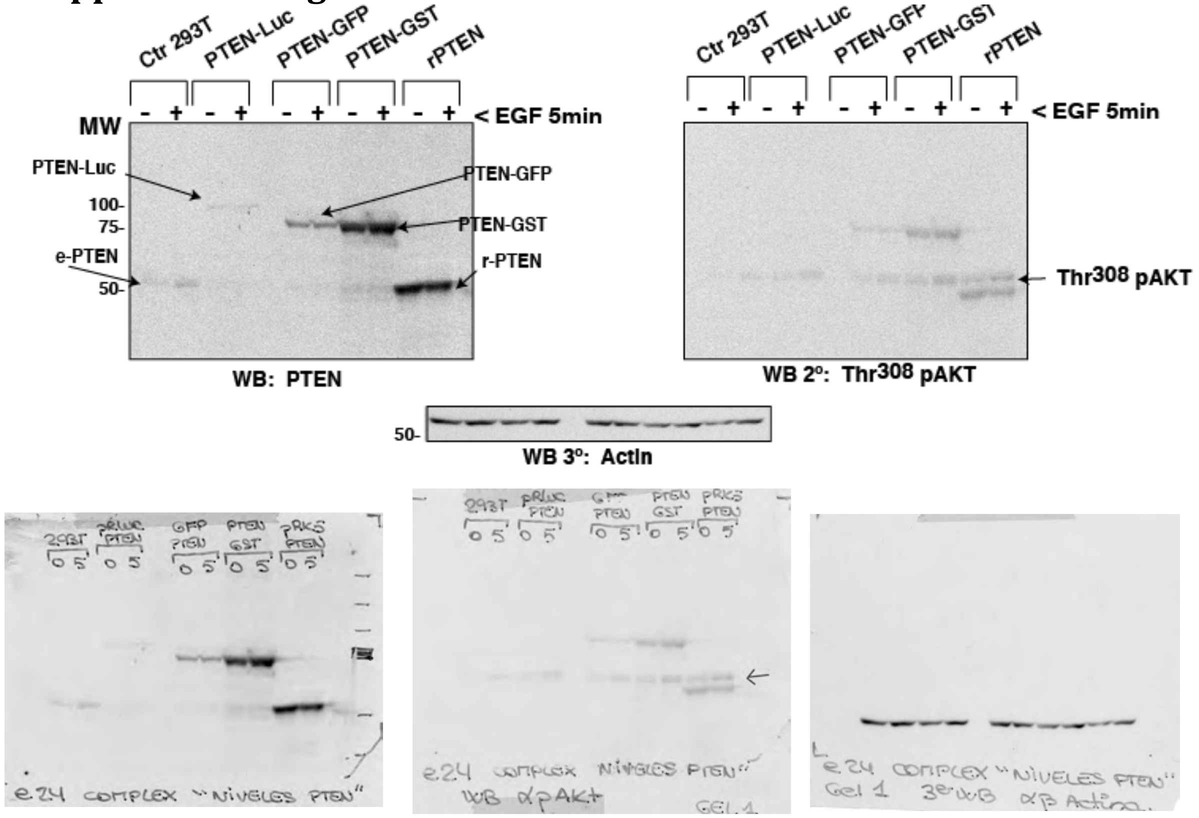

Supplemental FigS3

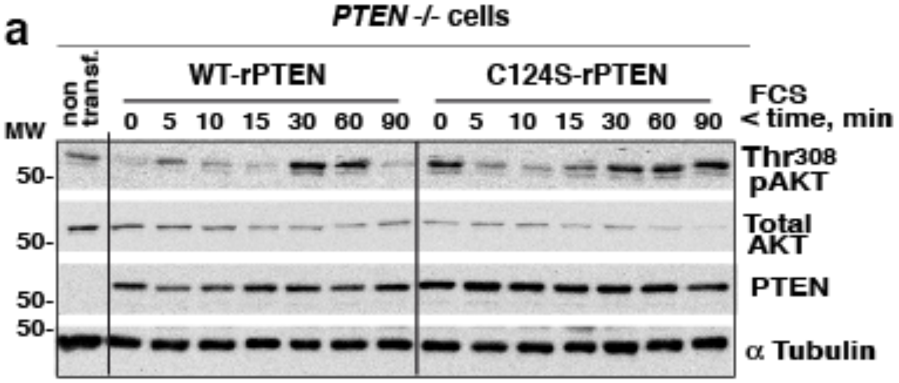

Thr308pAKT

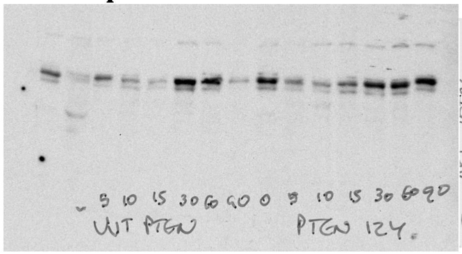

Total AKT

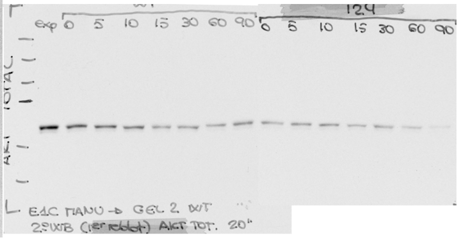

PTEN

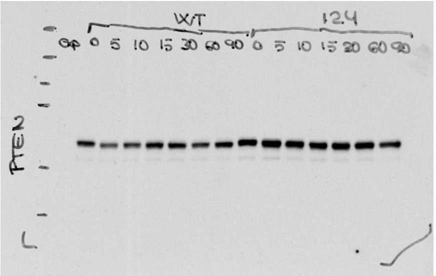

αTubulin

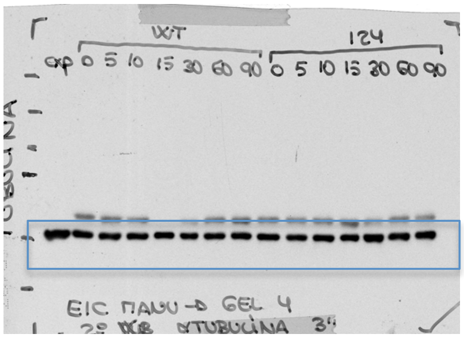

Supplemental FigS4a

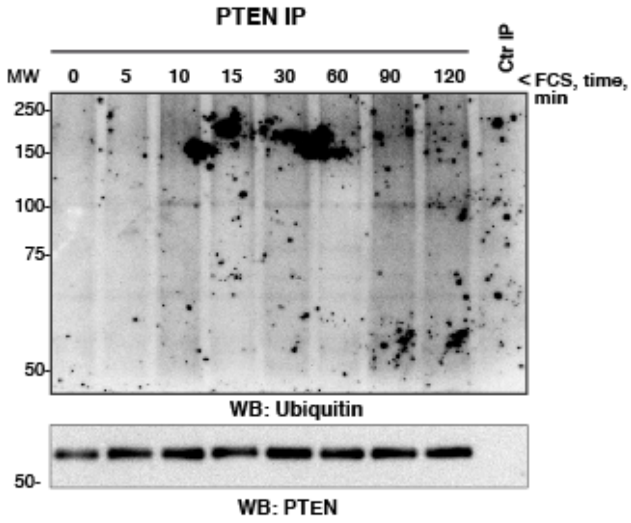

Ubiquitin WB

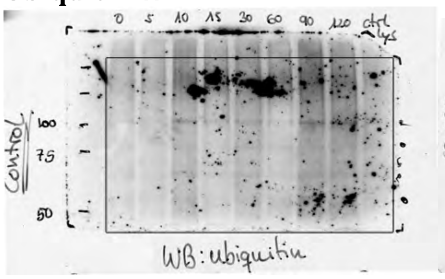

PTEN WB

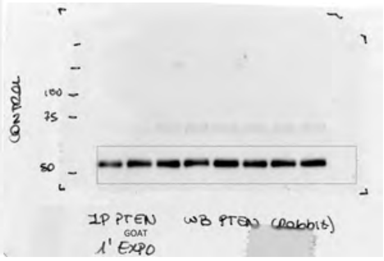

Supplemental FigS5a:

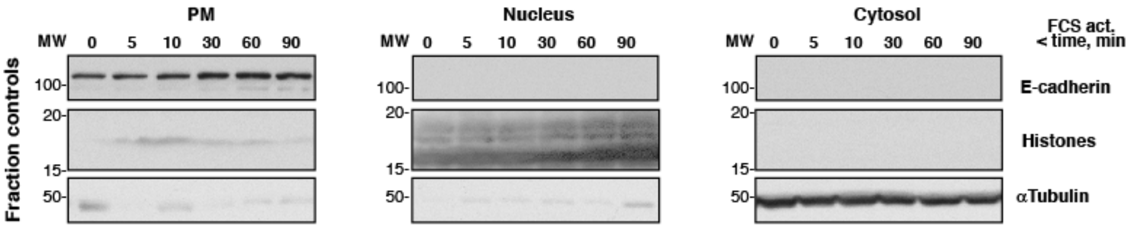

E-Cadherin

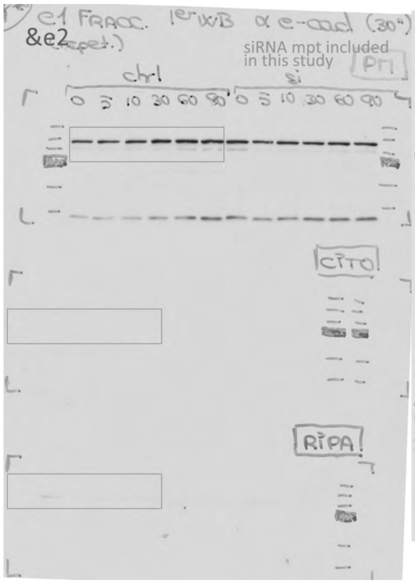

Histones

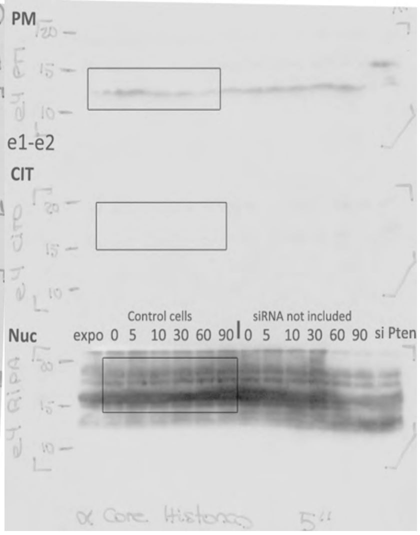

αTubulin

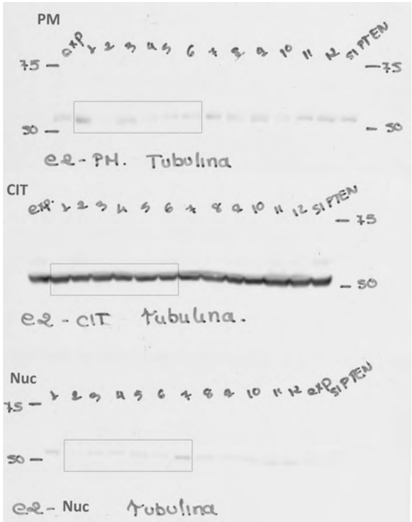

Supplemental FigS5b:

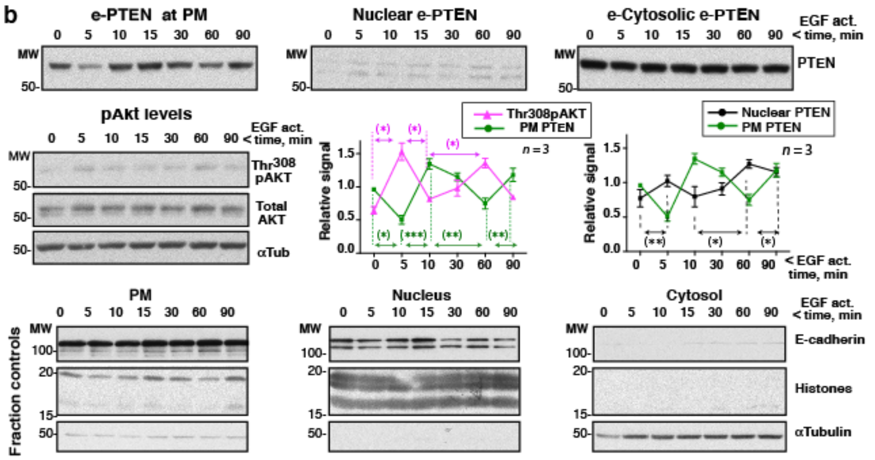

PTEN in different fractions   T308pAKT, Total AKT and Tubulin

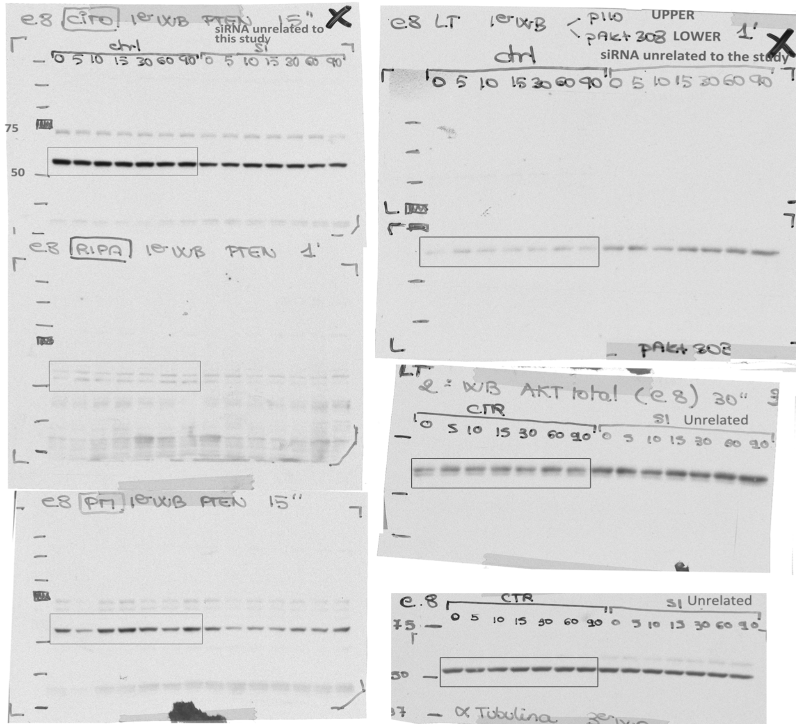

E-Cadherin, Histones and Tubulin controls in different fractions

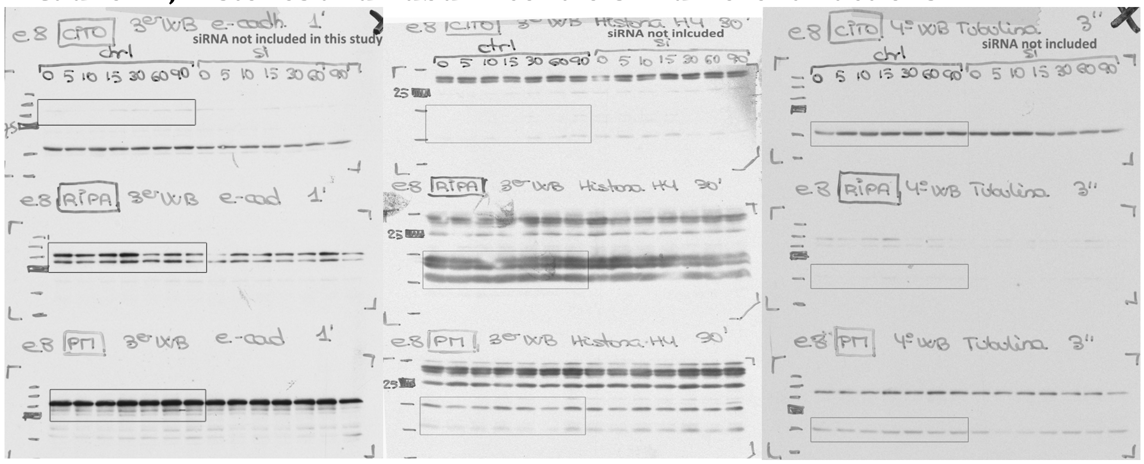

Supplemental FigS5c:

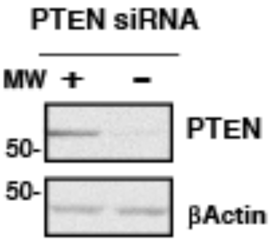

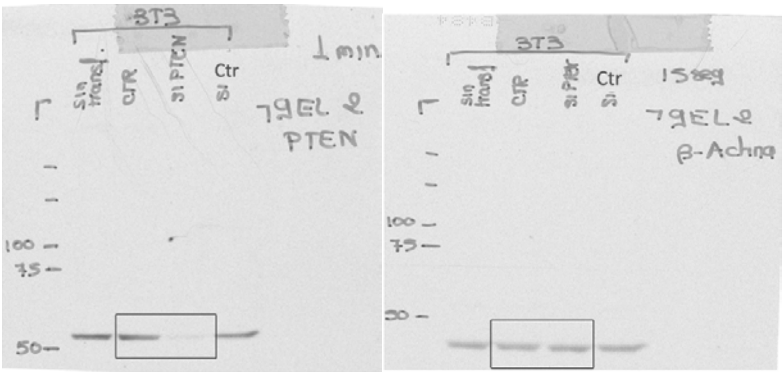

Supplemental Fig6Sa:

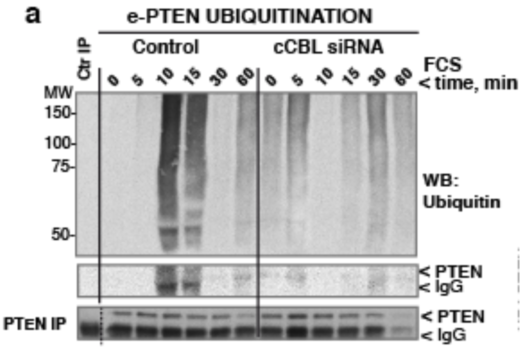

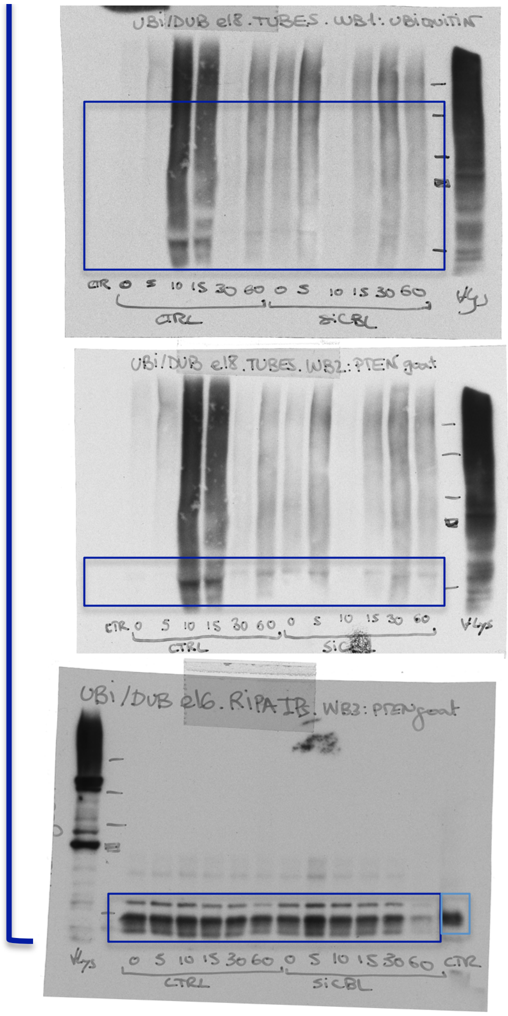

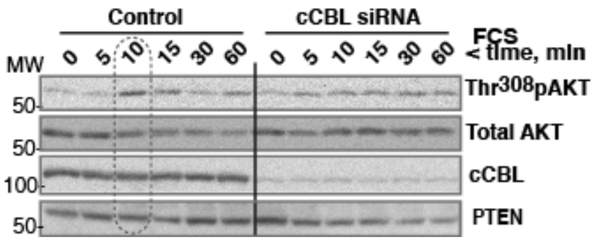

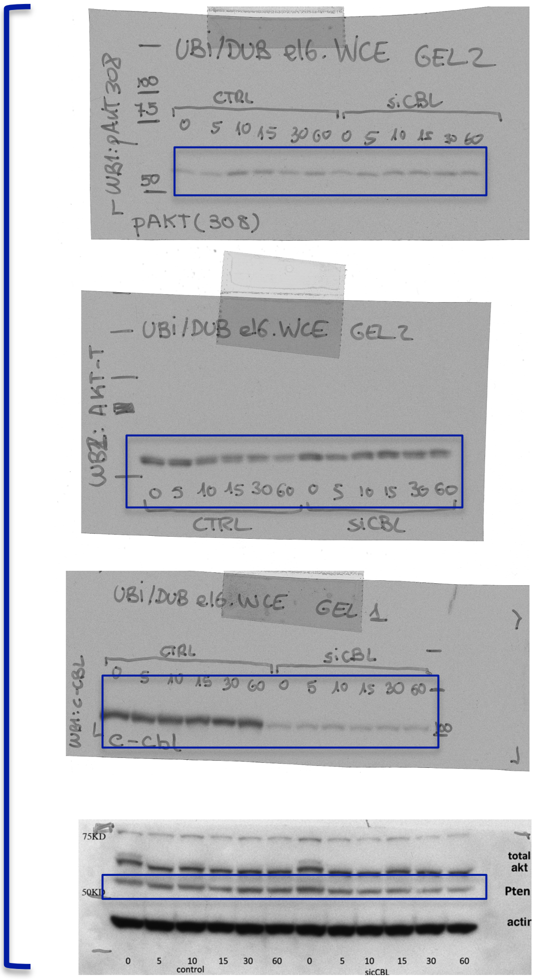

Supplemental Fig6b:

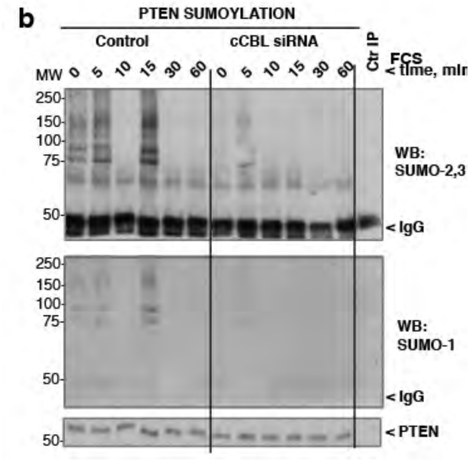

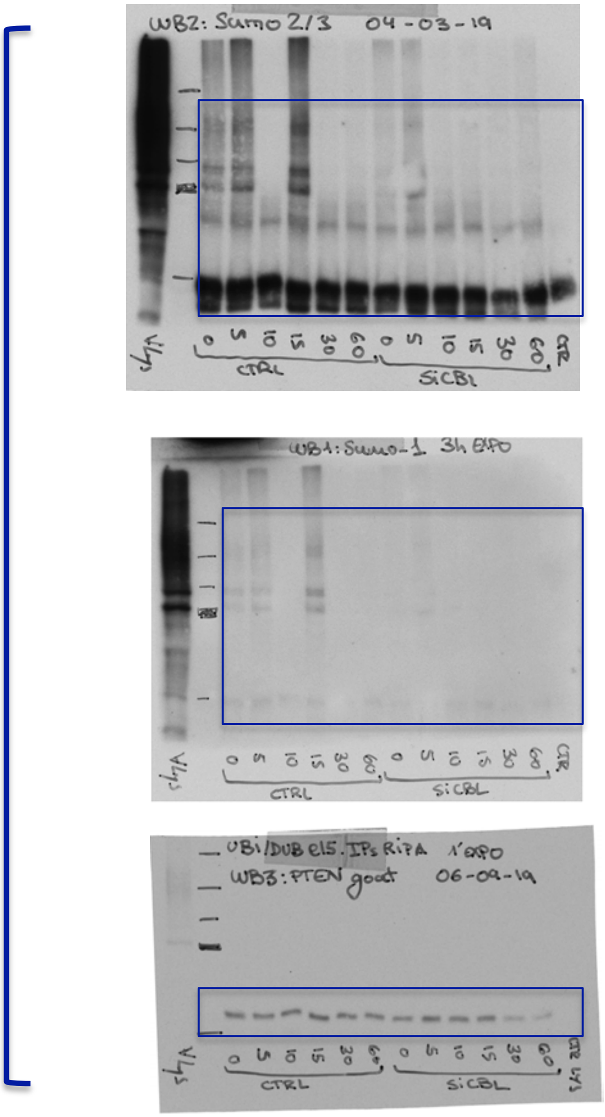

Supplemental Fig6c: in vitro ubi

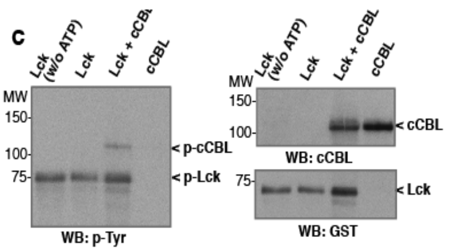

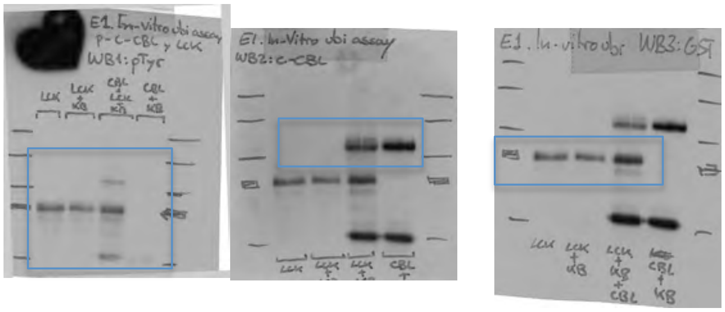

1º pTyr **2ºcCBL** **3º GST**

Supplemental Fig6d and e:

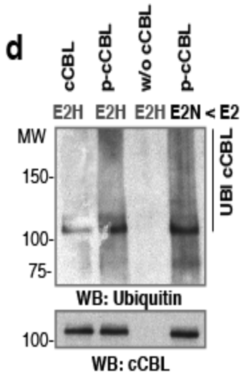

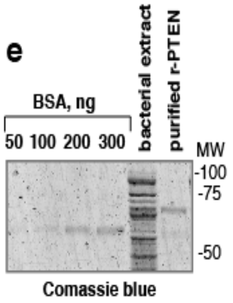

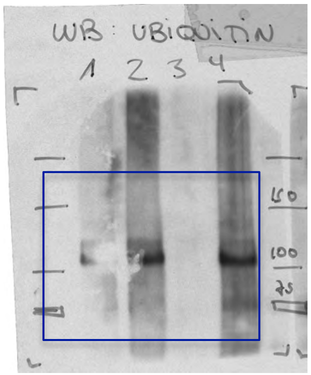

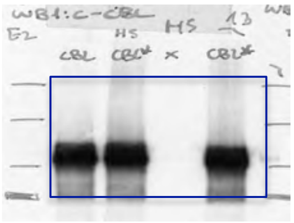

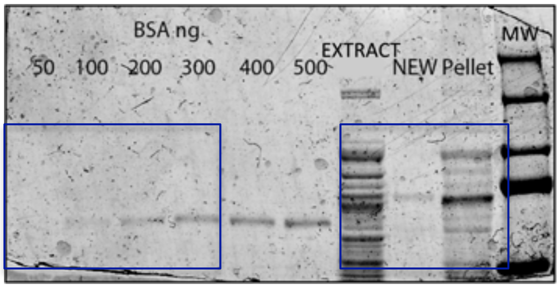

Supplemental Fig6f:

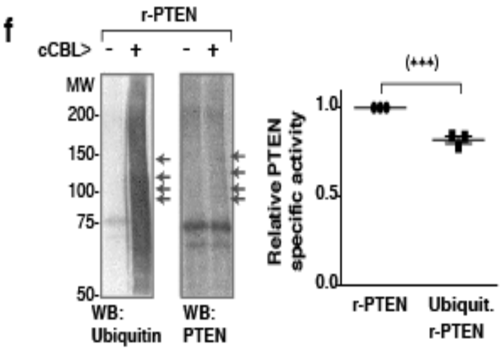

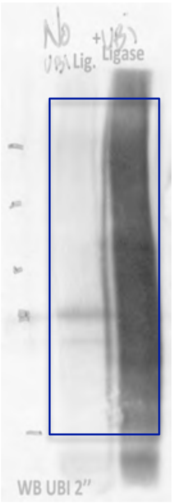

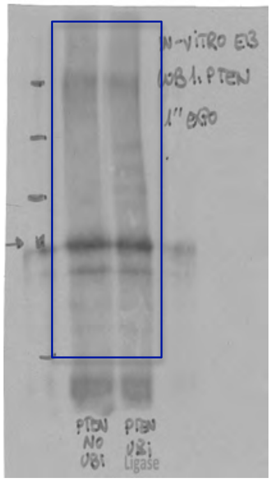

Figura S7A NO WB

Figura S7b WCE controls from e40 WCE and e47 WCE

e40 1stWB p85alpha (TOP) e40 2ºWB cCBL (TOP)

308pAKT (BOTTOM) TOTAL AKT (BOTTOM)

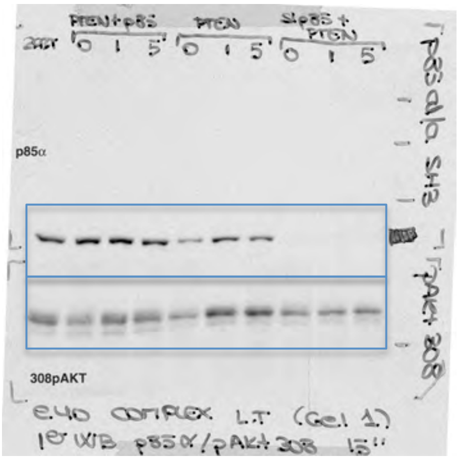

p85α

308 pAKT

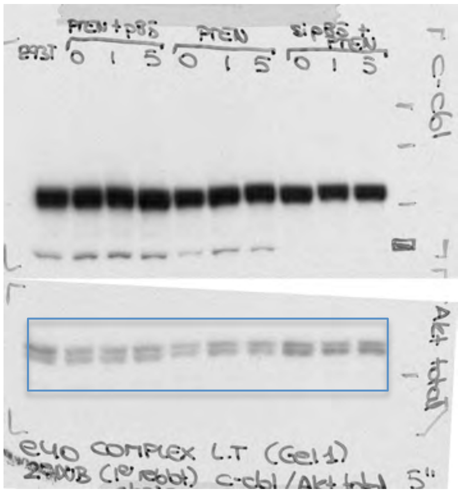

TOP NO USADO

Total AKT

e47 gel 3 1ºWB PTEN e47 gel 3 2ºWB cCBL

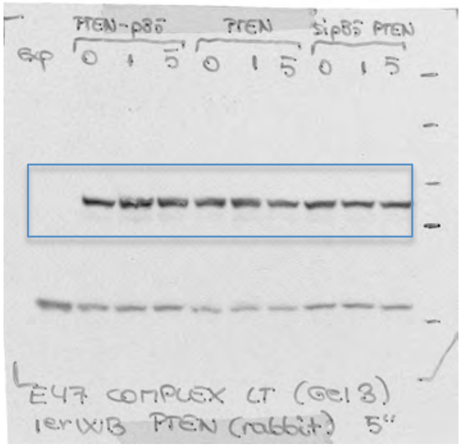

<r-PTEN

<e-PTEN

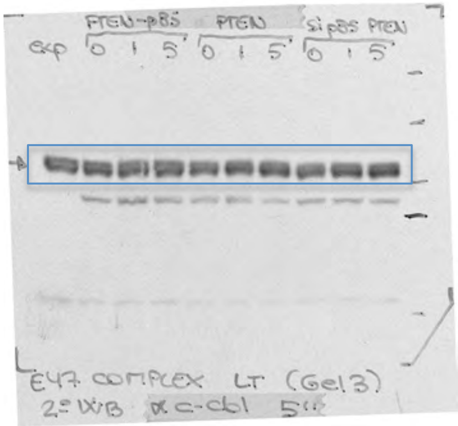

<cCBL

<r-PTEN (WB1º)

e47 gel 2 1ºWB p85b e47 gel 2 2ºWB p110β

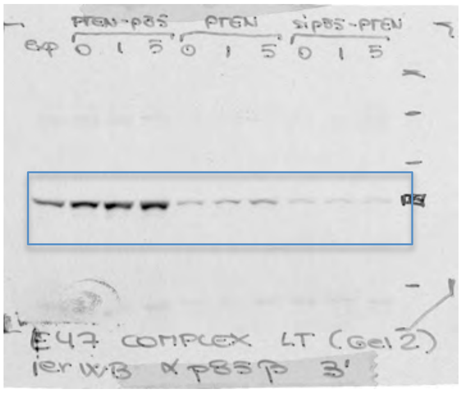

p85β

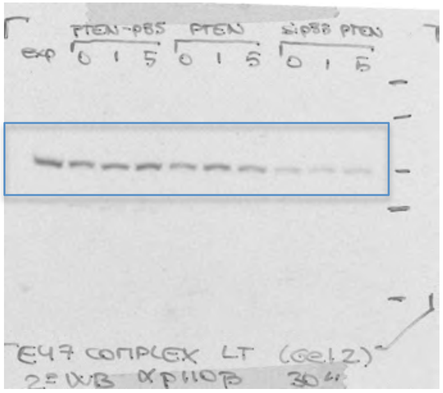

p110β

e47 gel 1 2ºWB p110a(TOP)

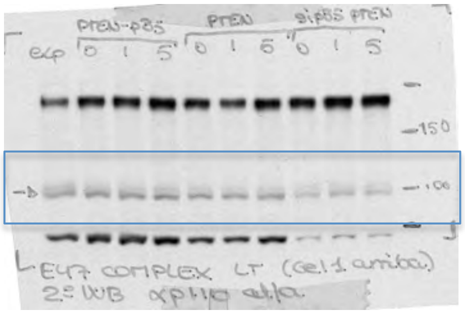

< non-specific band of p110α Ab

<p110α

<p85α (WB1º)

e47 gel 2 3ºWB EGFR

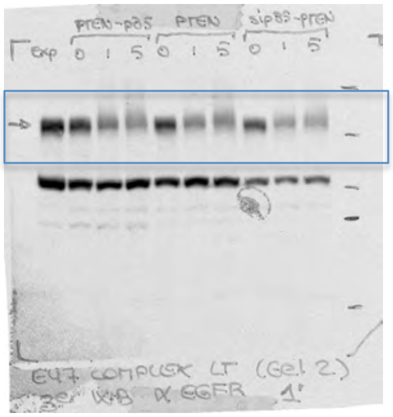

<EGFR

< p110β (2º WB)

e47 gel 3 3ºWB (after reblot) αTubulin

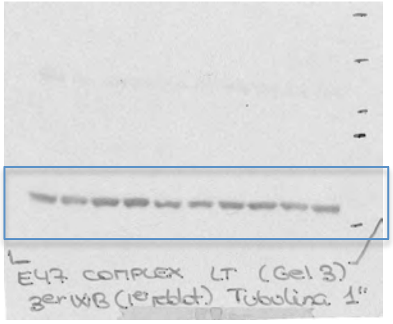

< αTubulin

Figura S7c EGFR IP, WB PTEN

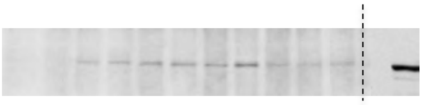

WB PTEN

same blot less exposed (for right lane)

75Kda>

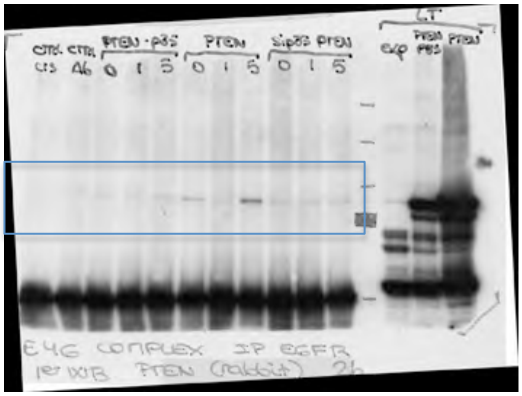

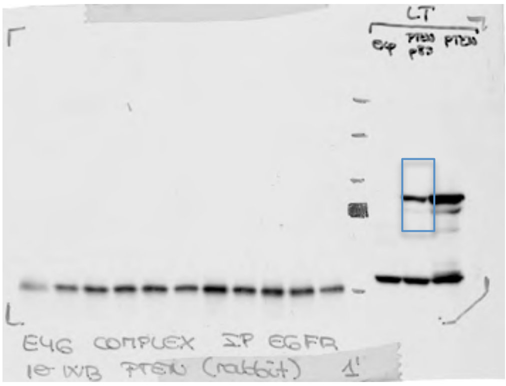

<r-PTEN

Figura S7c cCBL IP, WB PTEN

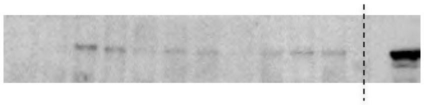

WB PTEN

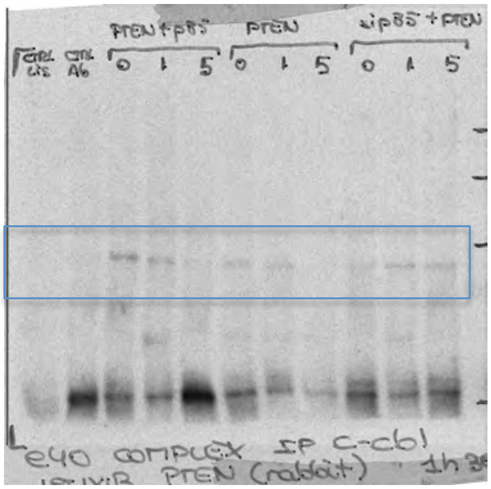

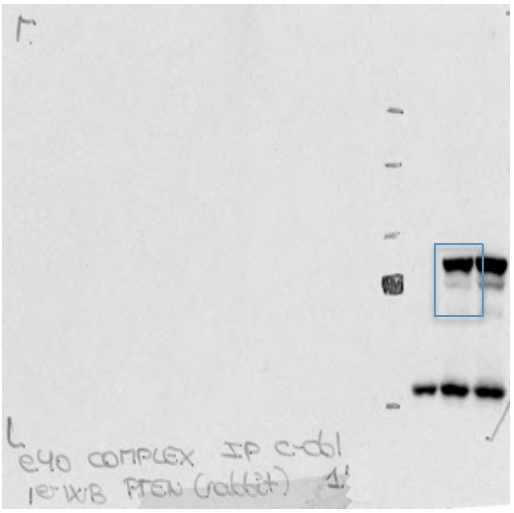

same blot less exposed (for last lane)

< r-PTEN

7ºpTyr

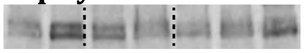

8ºpEGFR

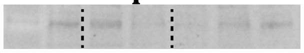

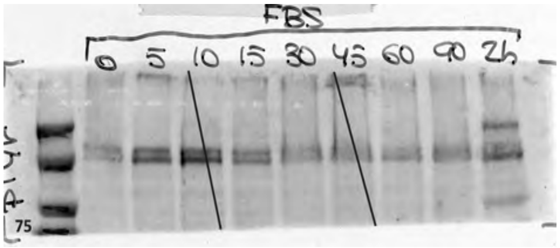

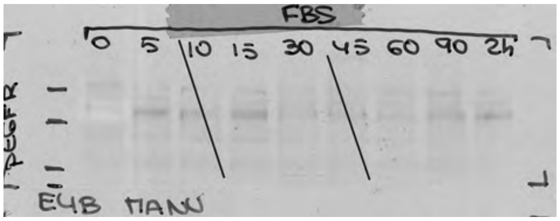

9º EGFR total

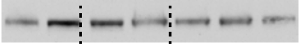

10º pERK

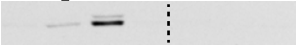

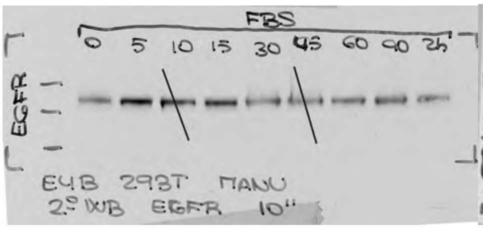

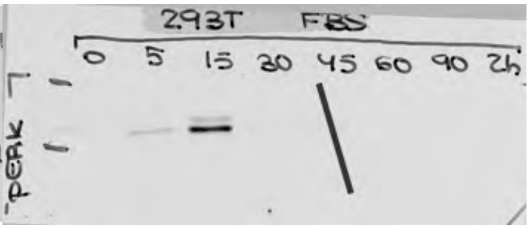

11º ERK total

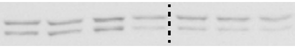

12º βActin

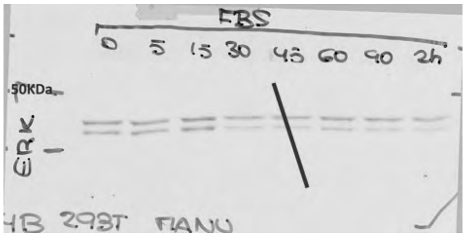

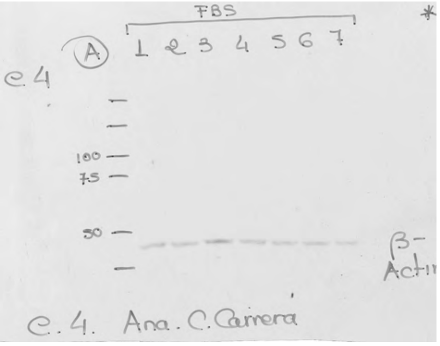

Fig1b: **PTEN IP and WB**

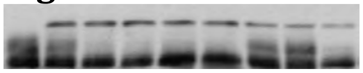

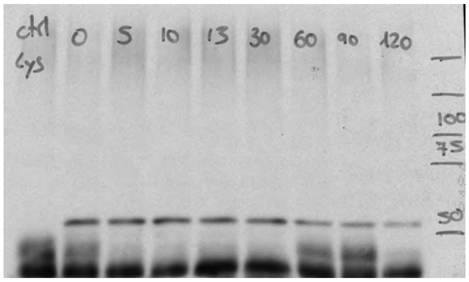

Fig1d:

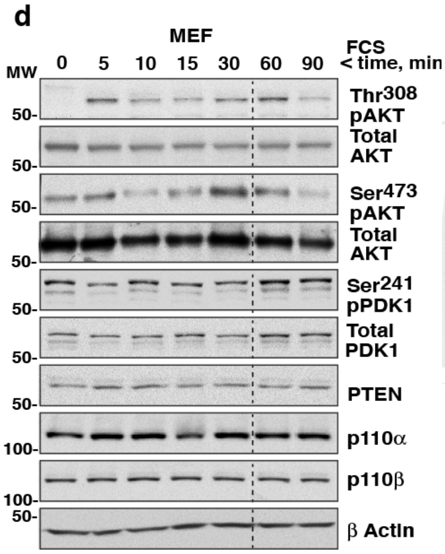

1ºT308 pAKT

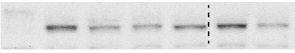

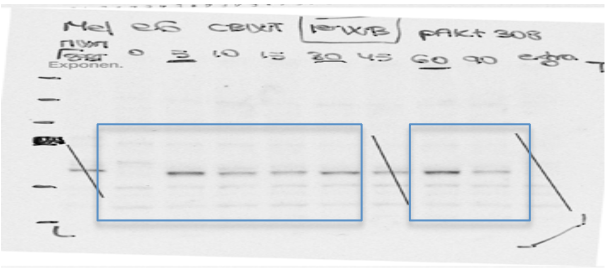

2ºTotal AKT

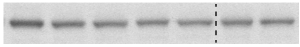

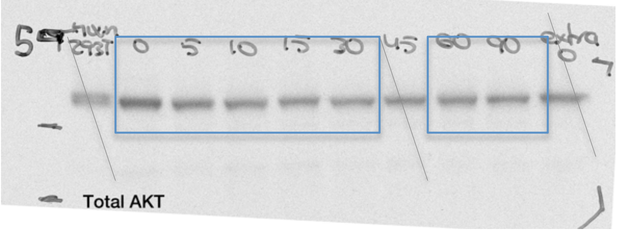

3ºS473 pAKT

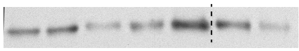

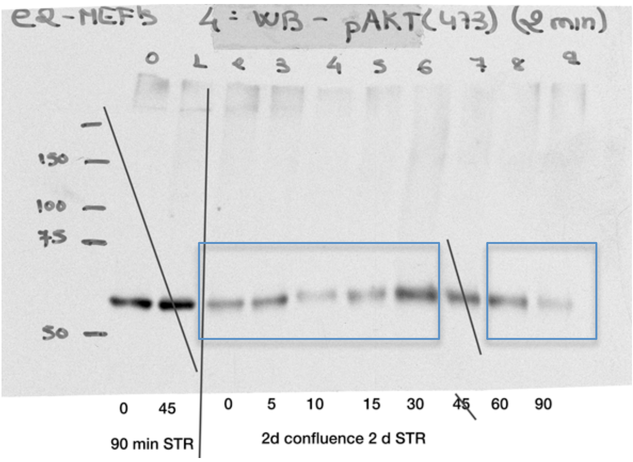

4ºTotal pAKT

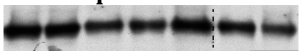

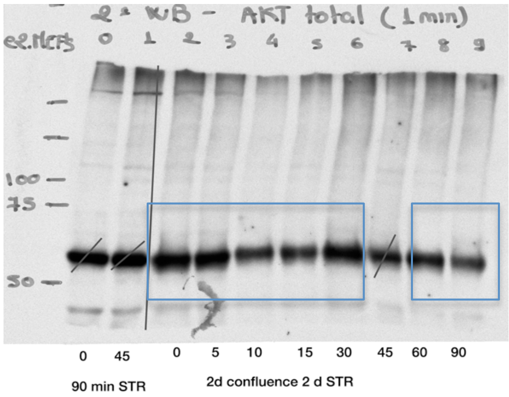

5º S241 pPDK1 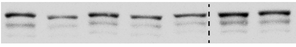

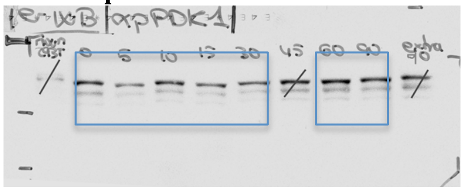

6º Total PDK1 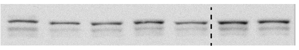

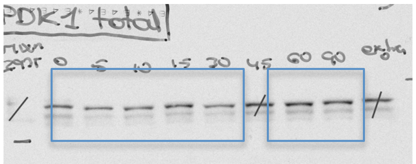

7º PTEN 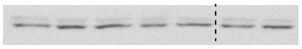

8º p110α 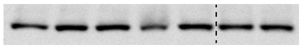

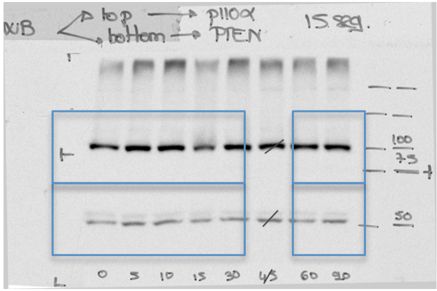

9º p110β 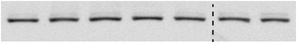

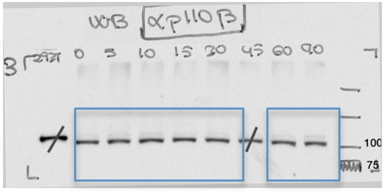

10º β Actin 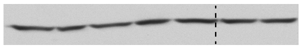

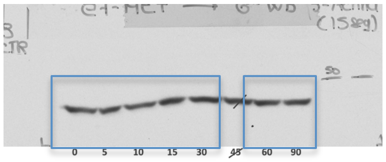

In these experiments several gels from the same samples had to be resolved to do all the presented blots.

Fig.3a:

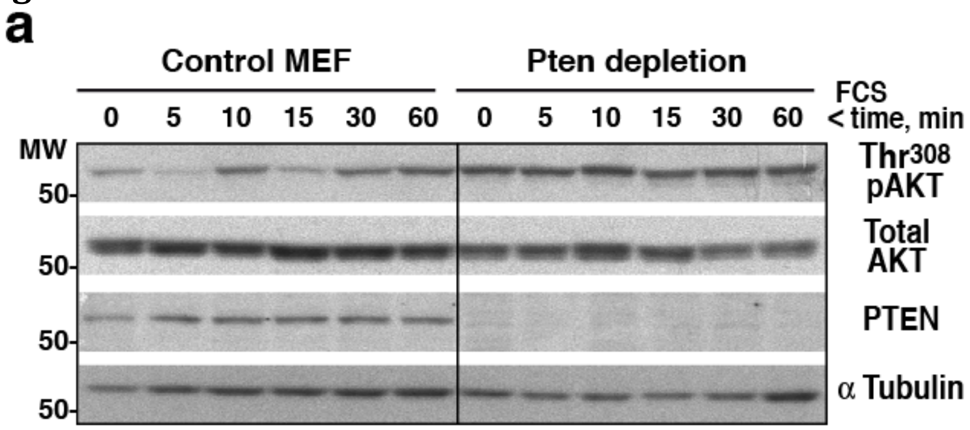

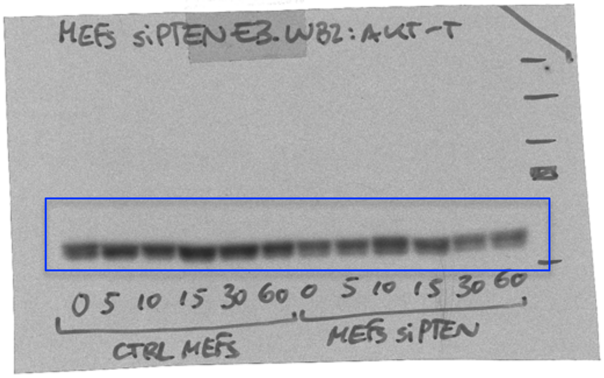

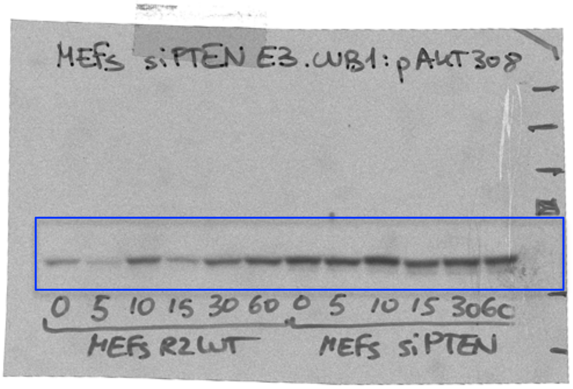

Tubulin in low section 75KDa-37KDa

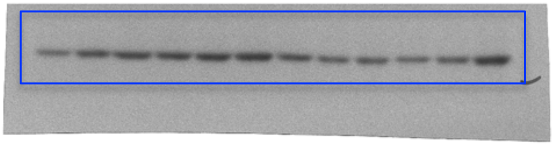

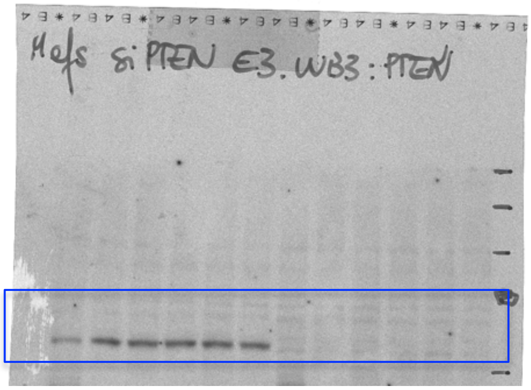

Fig.3b:

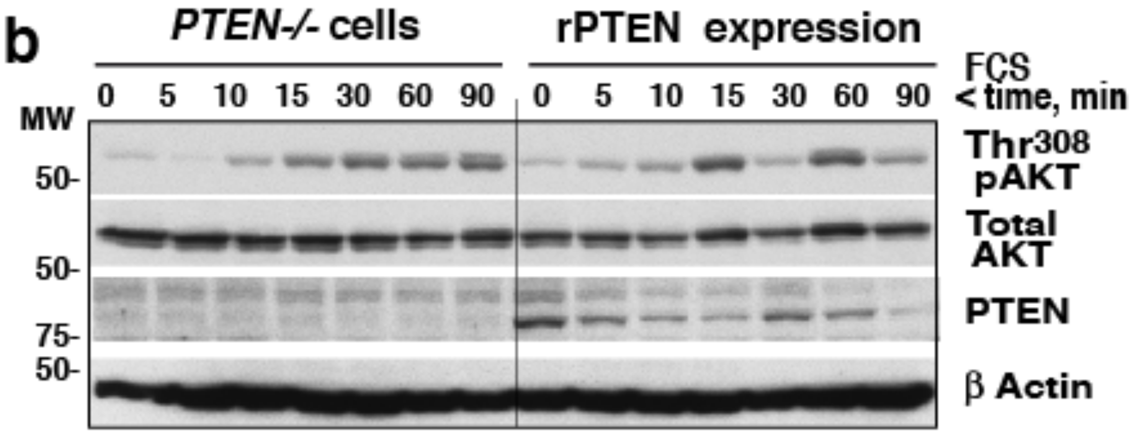

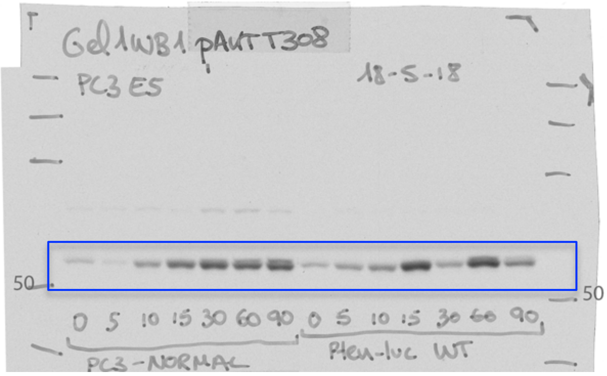

1º T308pAKT

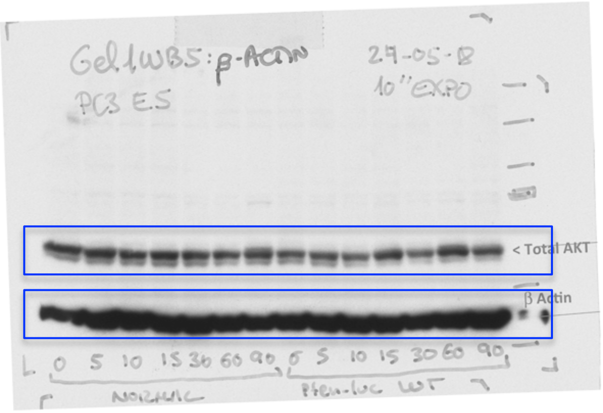

2º AKT

<<<<<4ºβ Actin

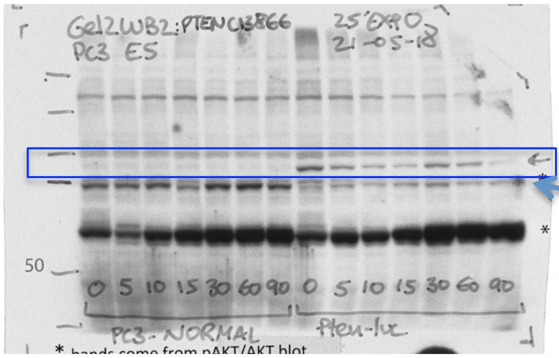

3ºPTEN

from 2º WB Total AKT

<<from 2º WB Total AKT

Fig.3b bottom:

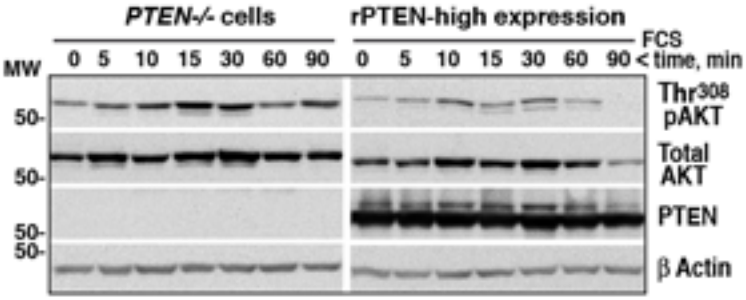

In all original gels Control_PTEN-/- cells are on the top

WB T308 pAKT **WB Total AKT**

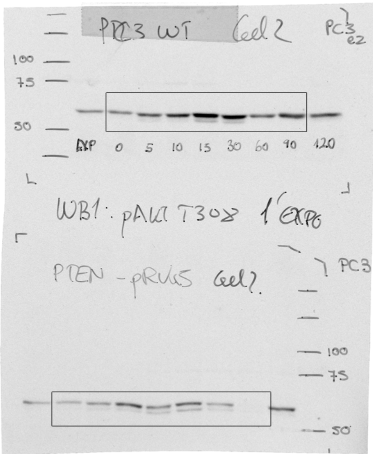

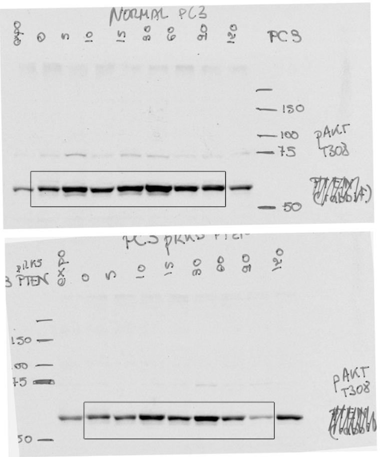

WB PTEN

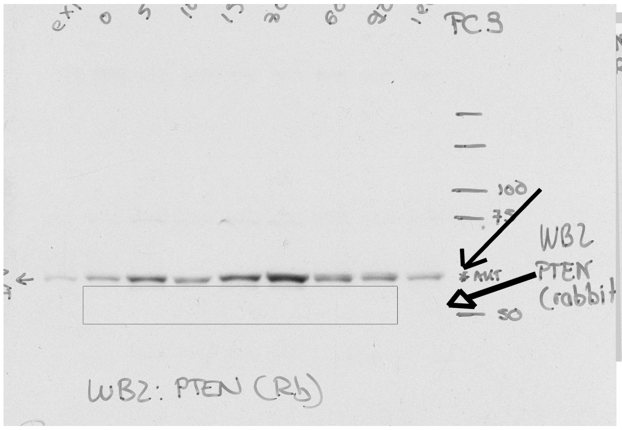

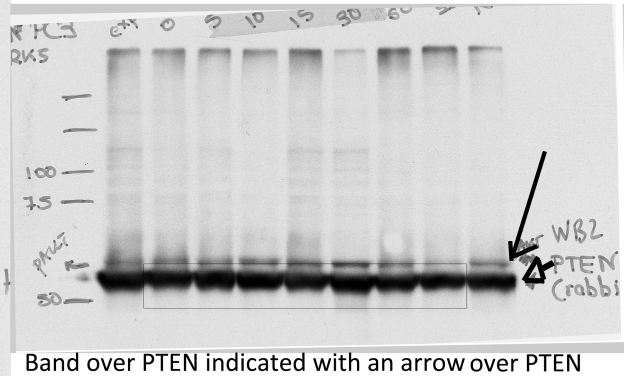

Band over PTEN indicated with an arrow over PTEN is total AKT from previous blot

WB ACTIN (samples resolved in a new gel)

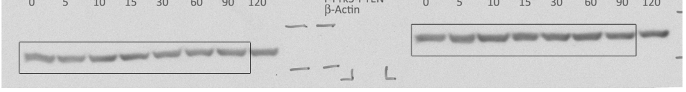

pRK5-PTEN Control PC3

Fig. 4a:

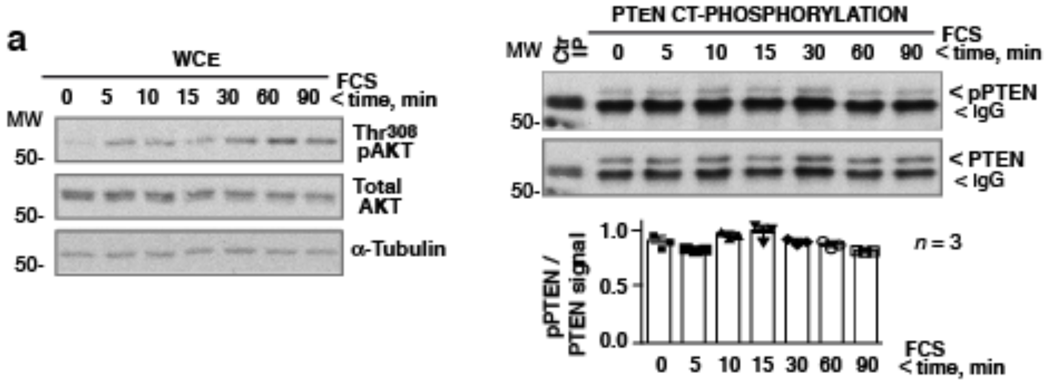

T308 p-AKT

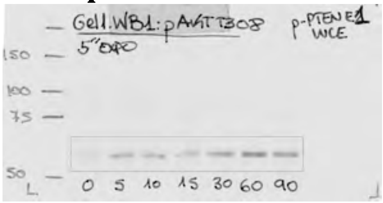

Total AKT

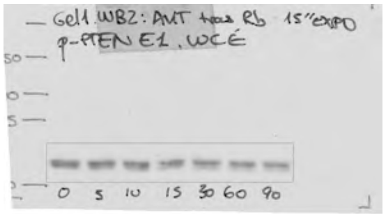

αTubulin

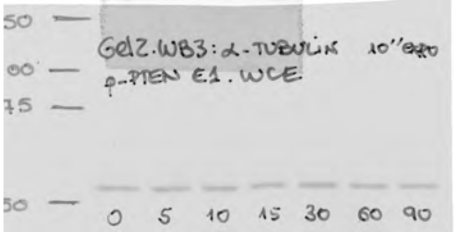

IP PTEN,WB: phospho-PTEN

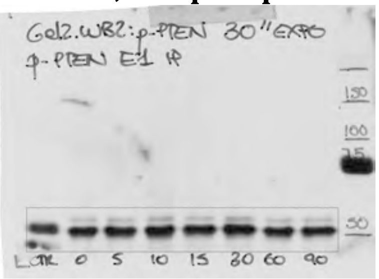

IP PTEN,WB: PTEN

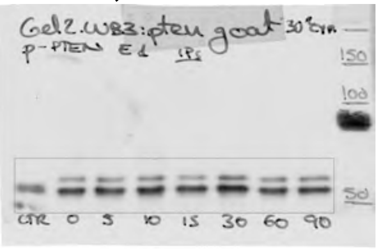

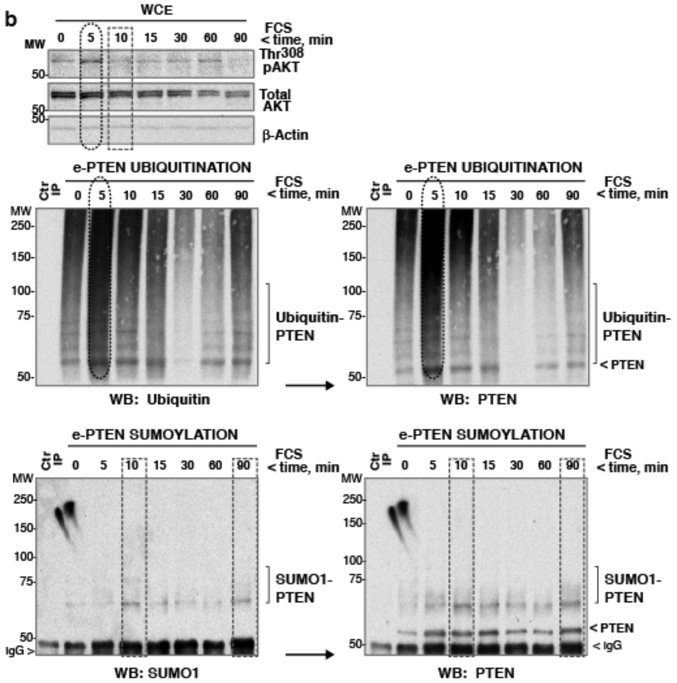

Fig. 4b:

T308pAKT

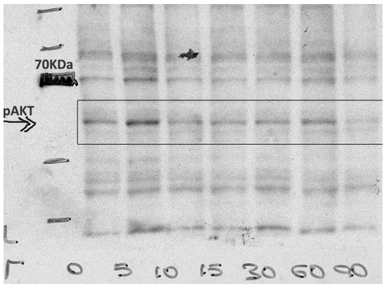

Total AKT

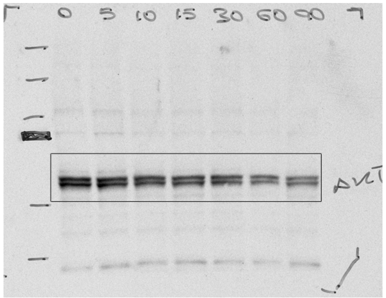

Actin

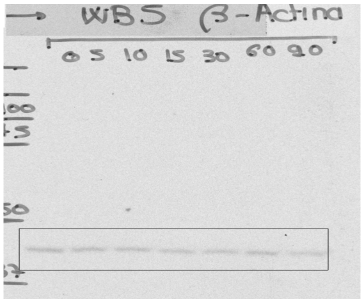

WB: UBI

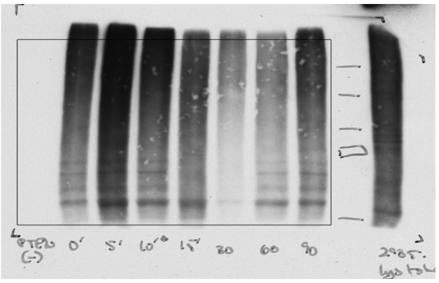

2ºWB PTEN

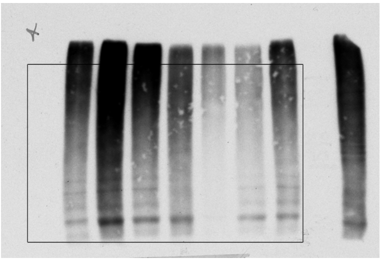

WB: SUMO1

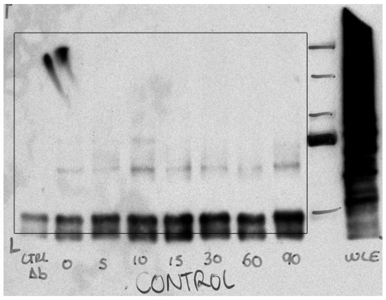

2ºWB PTEN

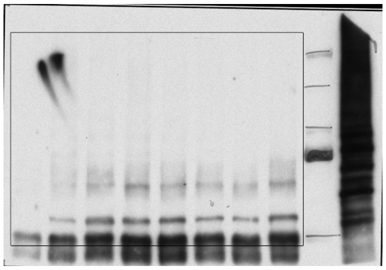

Fig5a:

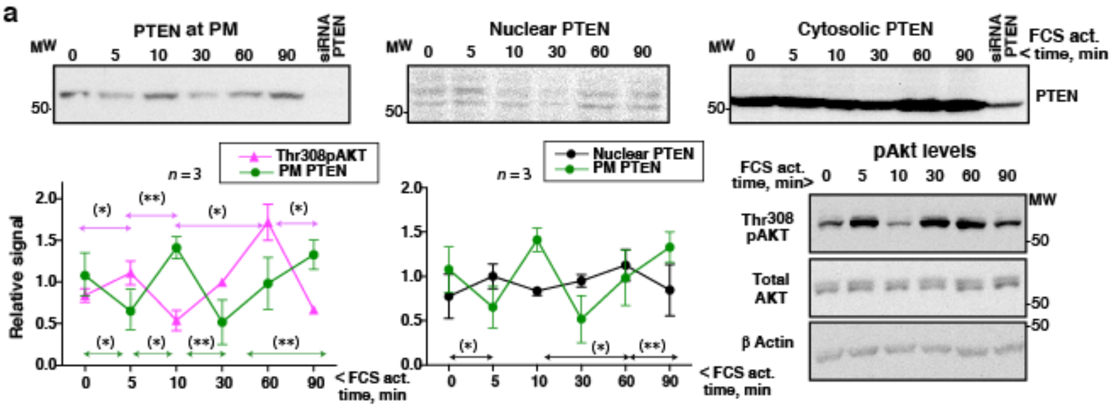

PTEN PM

PTEN Nucleus

PTEN Citosol

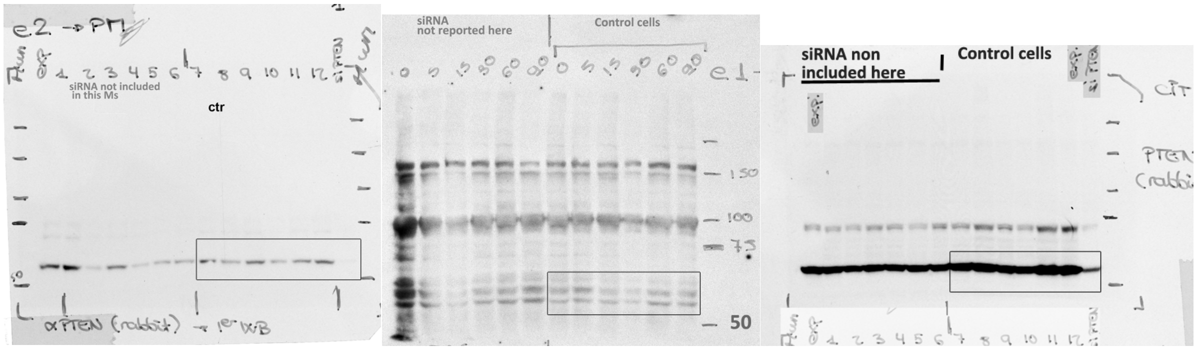

T308AKT

Total AKT

β-Actin

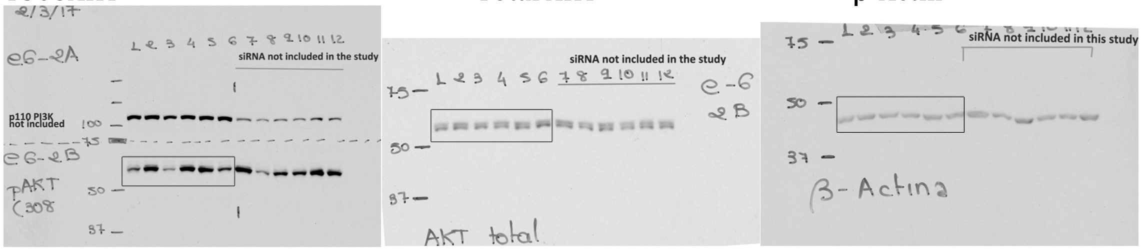

Fig5b:

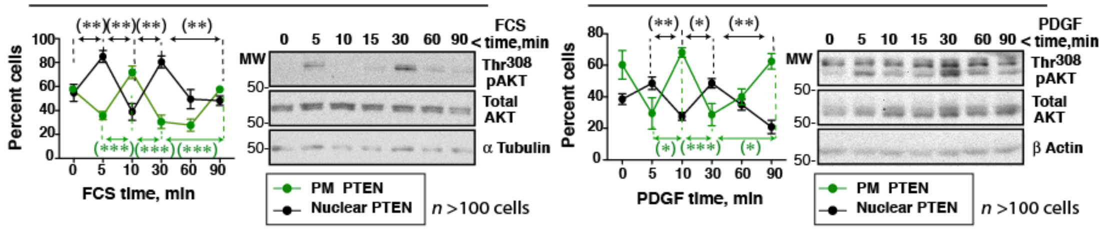

FCS T308AKT

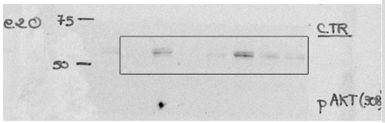

Total AKT

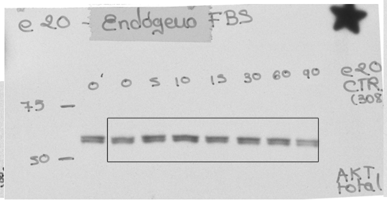

α-Tubulin

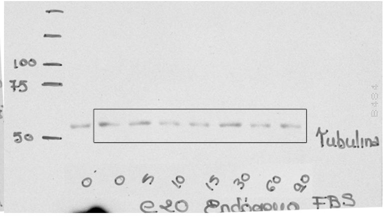

PDGF T308AKT

note that in these blots times go from right to left

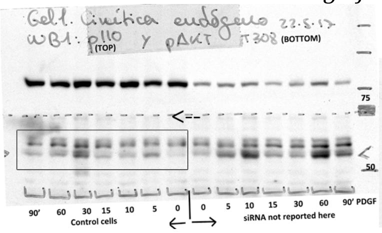

Total AKT

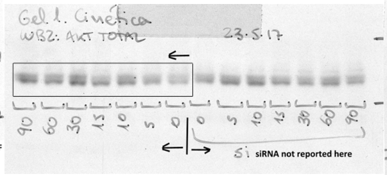

β-Actin

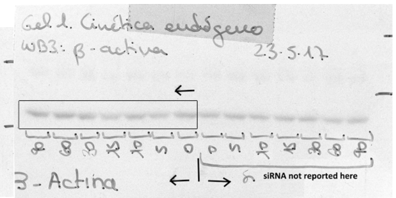

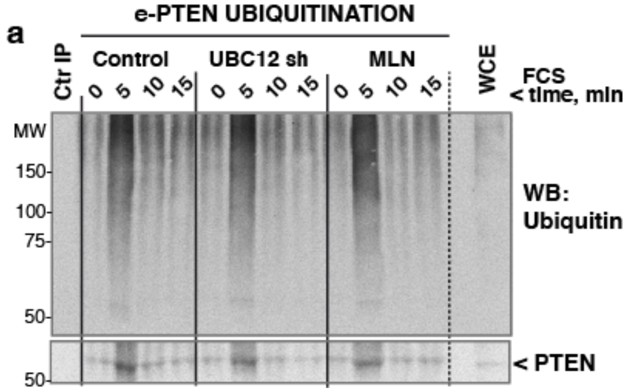

Figure 6a

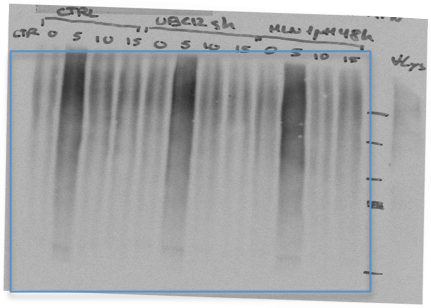

Ubiquitin

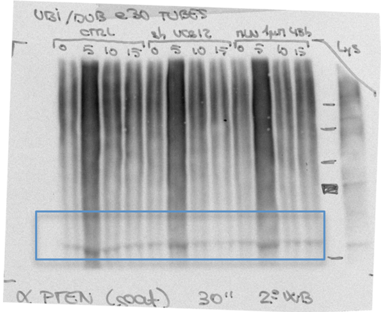

<PTEN

CONTROL MLN

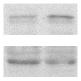

p27

αTubulin

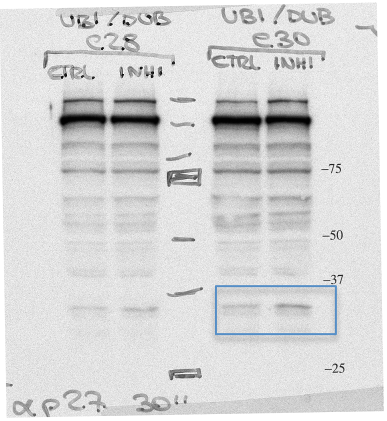

<p27

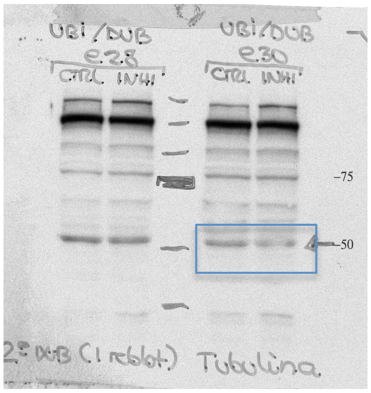

<αTubulin

WCE WB

Thr308pAKT

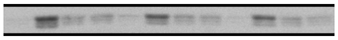

TOTAL AKT

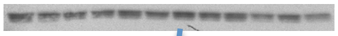

αTubulin

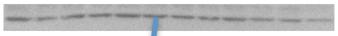

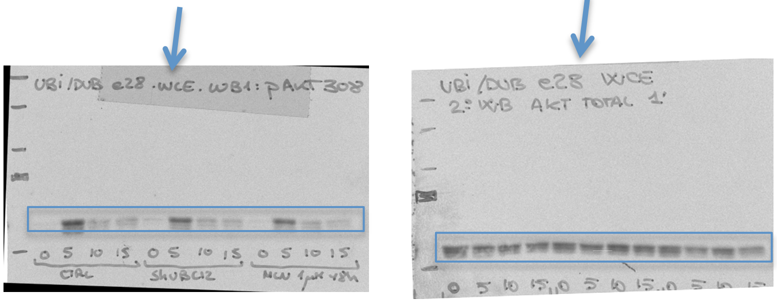

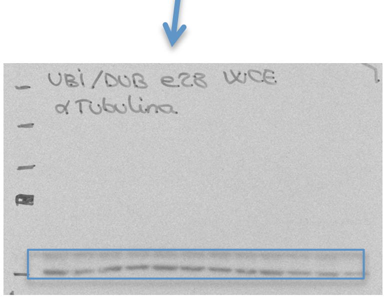

Figure 6b

PTEN IP, 1st WB Ubiquitin

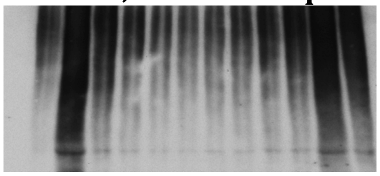

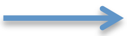

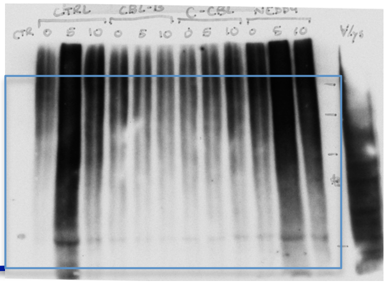

siRNA CONTROLS

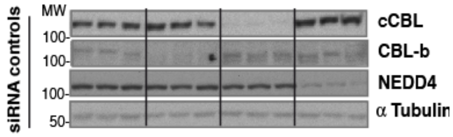

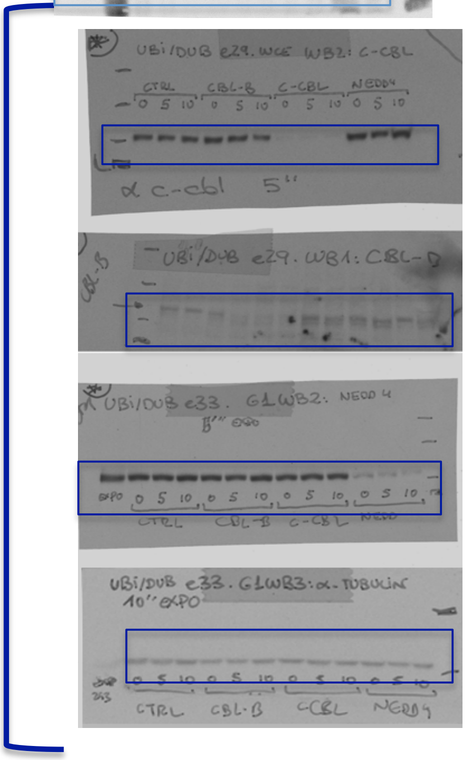

pAKT PTEN CONTROLS

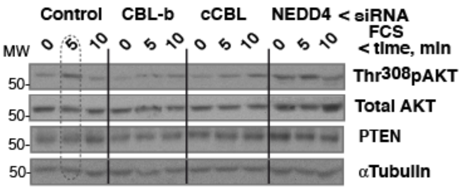

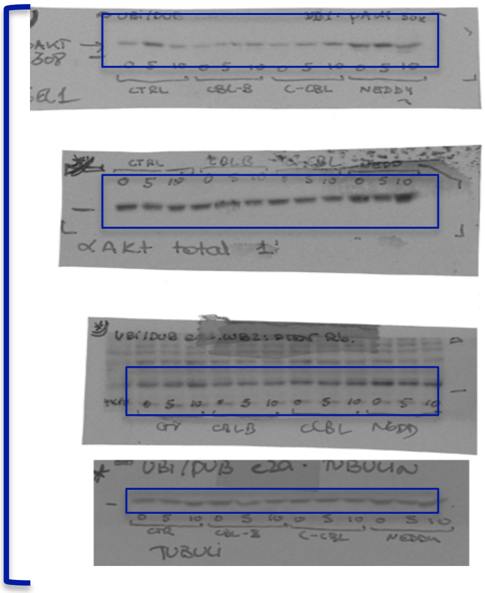

Figure 6c in vitro Ubi experiments

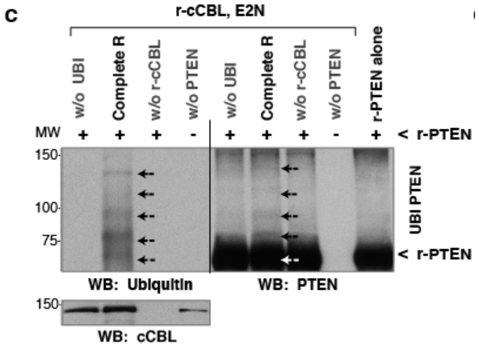

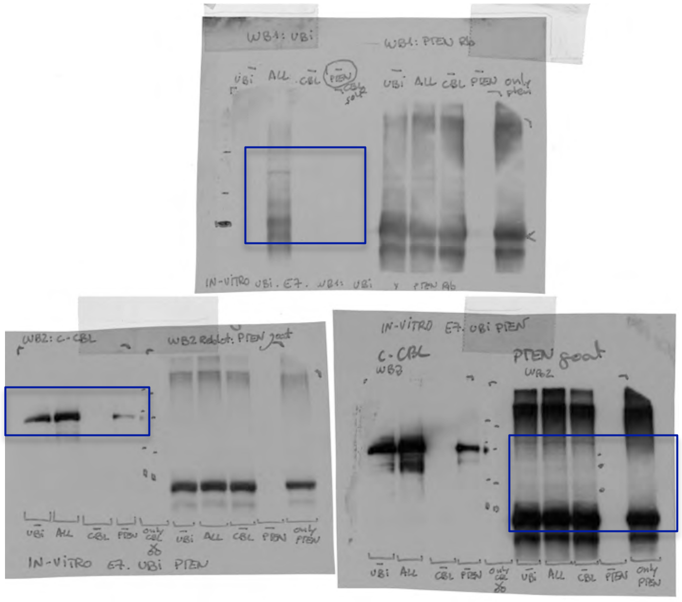

Figure 6d in vitro Ubi experiments

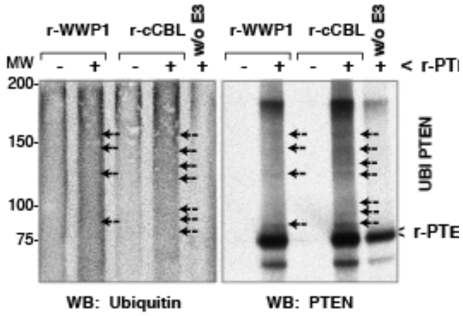

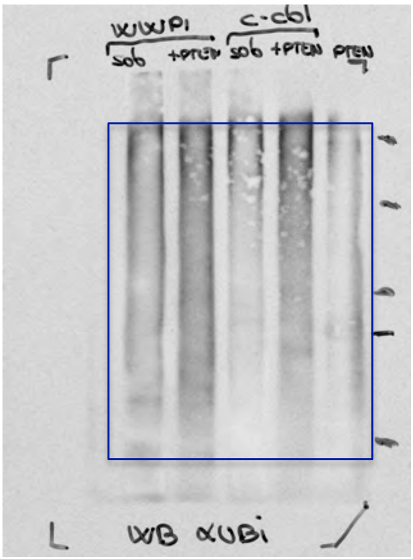

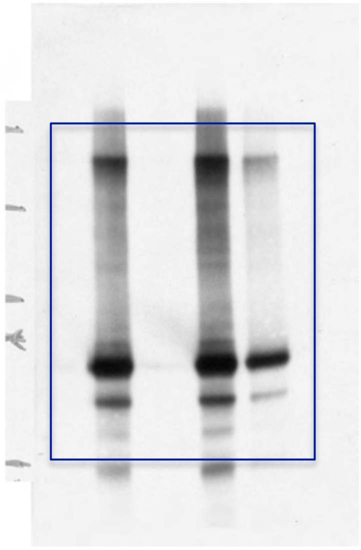

Fig. 7a LEFT **<u>IP EGFR</u>** WB PTEN and EGFR (LT = Total Lysate)

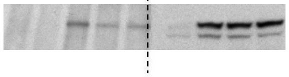

WB PTEN

2ºWB PTEN (after cCBL)

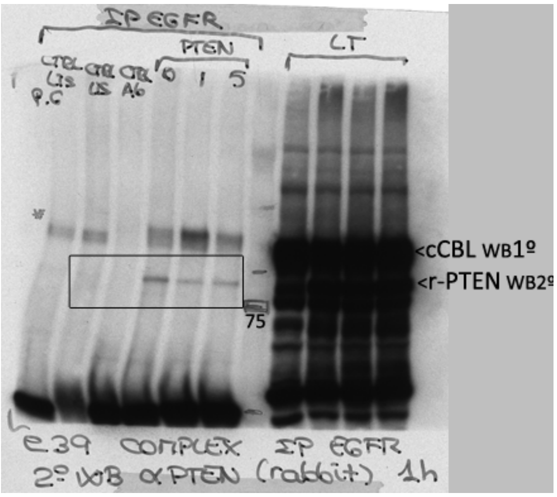

SAME GEL less exposed (for right lanes)

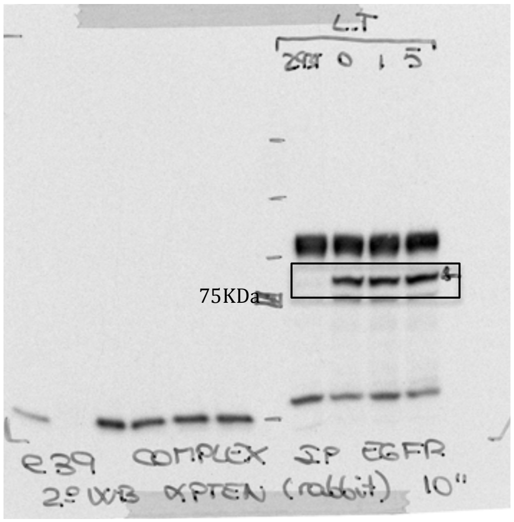

< cCBL (1º WB)

< **r-PTEN (2º WB)**

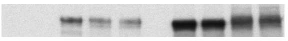

WB EGFR

3ºWB EGFR

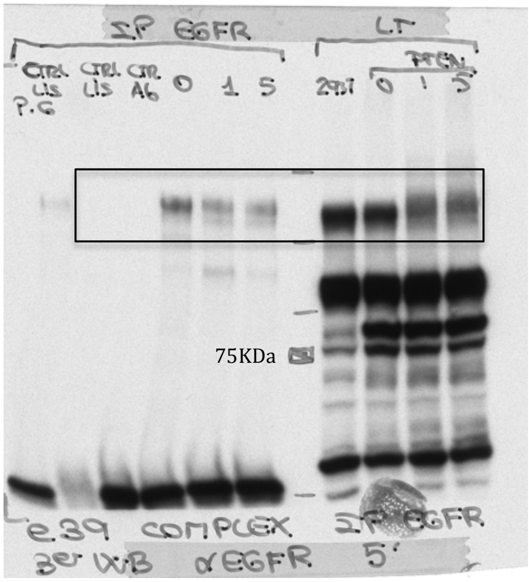

< **EGFR 3ºWB**

< cCBL (1º WB)

< r-PTEN(2º WB)

Fig. 7a CENTRAL **cCBL IP** WB EGFR and WB cCBL

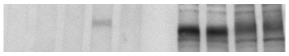

WB EGFR

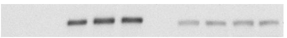

WB cCBL

2ºWB EGFR (after PTEN WB)

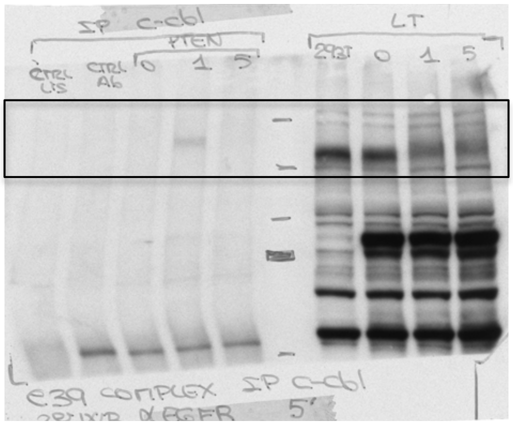

< EGFR
(2ºWB)

< r-PTEN>
(1ºWB)

3ºWB cCBL

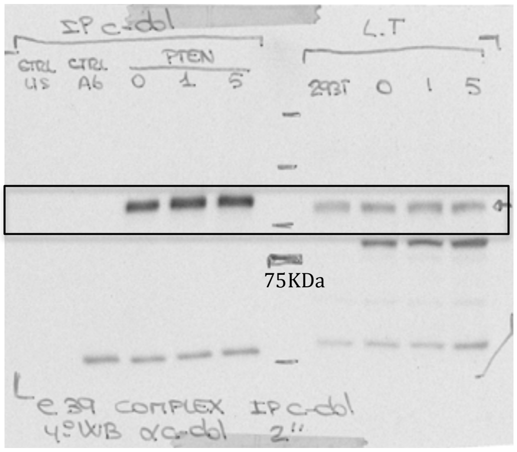

cCBL
3ºWB

Fig. 7a RIGHT **cCBL IP** WB PTEN and WB cCBL

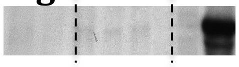

WB PTEN

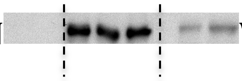

WB cCBL

1ºWB PTEN

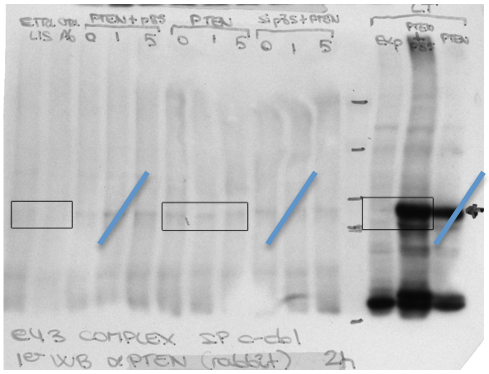

2ºWB cCBL

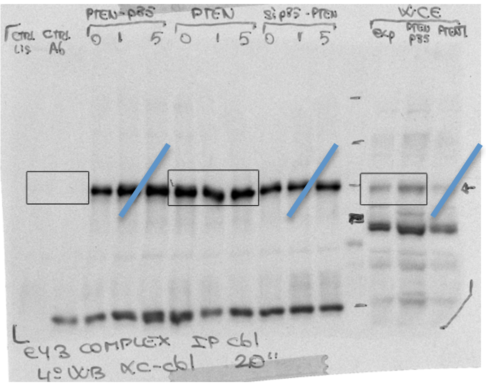

2ºWB cCB

Fig. 7b <u>**cCBL IP**</u> WB EGFR and cCBL

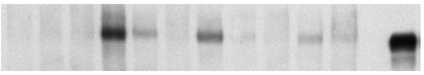

WB EGFR

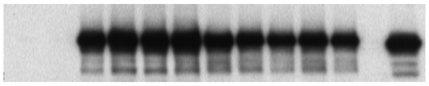

WB cCBL

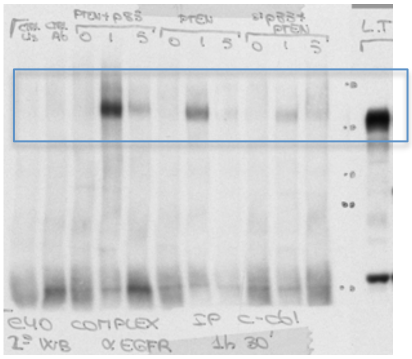

< 1ºWB **EGFR**

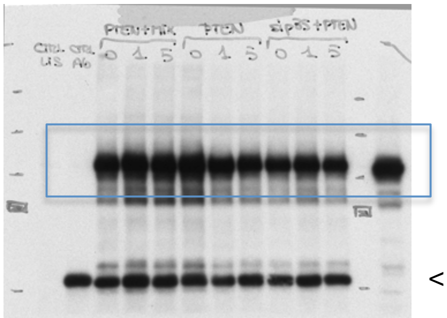

< 2ºWB **cCBL**
WB after reblot
<IgG

Fig. 7b <u>**EGFR IP**</u> WB cCBL and EGFR

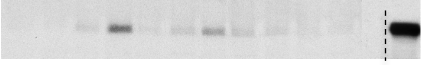

WB cCBL

2ºWB cCBL (after PTEN)

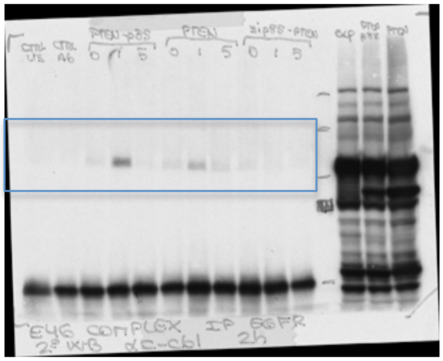

< 2ºWB **cCBL**
<1ºWB r-PTEN

SAME GEL SHORT EXPOSURE

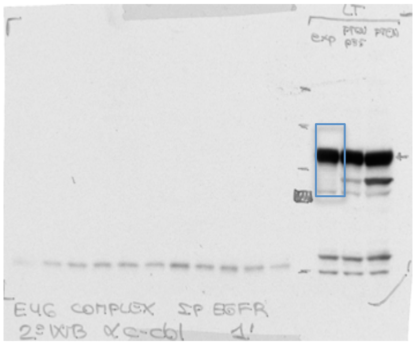

<2ºWB **cCBL**
<1ºWB r-PTEN

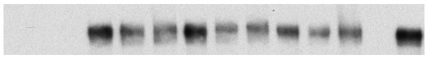

WB EGFR

3ºWB EGFR

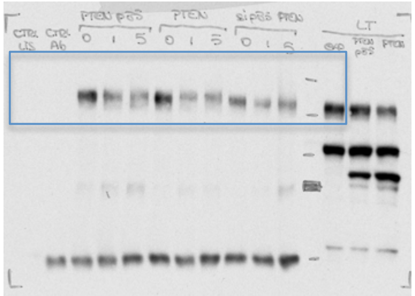

< 3ºWB **EGFR**
<2ºWB cCBL prev.
<1ºWB r-PTEN prev.

Figura 7e

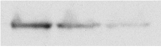

WB cCBL

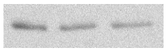

WB α Tubulin

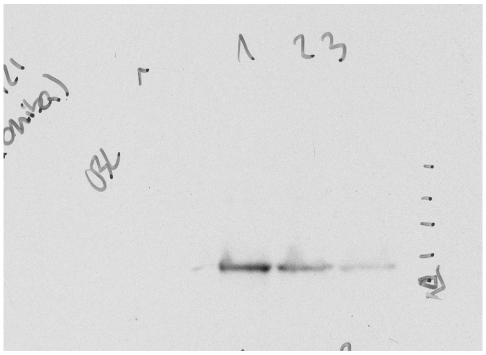

cCBL

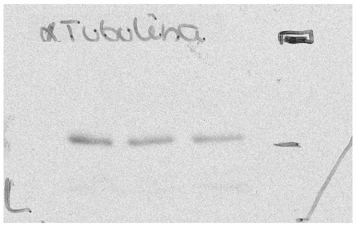

α Tubulin
